# Functioning of Microsomal Cytochrome P450s: *Murburn* Concept Explains the Metabolism of Xenobiotics in Hepatocytes

**DOI:** 10.3389/fphar.2016.00161

**Published:** 2016-06-23

**Authors:** Kelath Murali Manoj, Abhinav Parashar, Sudeep K. Gade, Avanthika Venkatachalam

**Affiliations:** ^1^Satyamjayatu: The Science & Ethics FoundationKulappully, India; ^2^Hemoproteins Lab, School of Bio Sciences and Technology, VIT UniversityVellore, India; ^3^REDOx Lab, PSG Institute of Advanced StudiesCoimbatore, India

**Keywords:** cytochrome P450, *murburn*, coupling, activation, redox, xenobiotic/drug metabolism, electron transfer, reductase

## Abstract

Using oxygen and NADPH, the redox enzymes cytochrome P450 (CYP) and its reductase (CPR) work in tandem to carry out the phase I metabolism of a vast majority of drugs and xenobiotics. As per the erstwhile understanding of the catalytic cycle, binding of the substrate to CYP's heme distal pocket allows CPR to pump electrons through a CPR-CYP complex. In turn, this trigger (a thermodynamic push of electrons) leads to the activation of oxygen at CYP's heme-center, to give Compound I, a two-electron deficient enzyme reactive intermediate. The formation of diffusible radicals and reactive oxygen species (DROS, hitherto considered an undesired facet of the system) was attributed to the heme-center. Recently, we had challenged these perceptions and proposed the *murburn* (“mured burning” or “mild unrestricted burning”) concept to explain heme enzymes' catalytic mechanism, electron-transfer phenomena and the regulation of redox equivalents' consumption. *Murburn* concept incorporates a one-electron paradigm, advocating obligatory roles for DROS. The new understanding does not call for high-affinity substrate-binding at the heme distal pocket of the CYP (the first and the most crucial step of the erstwhile paradigm) or CYP-CPR protein-protein complexations (the operational backbone of the erstwhile cycle). Herein, the dynamics of reduced nicotinamide nucleotides' consumption, peroxide formation and depletion, product(s) formation, etc. was investigated with various controls, by altering reaction variables, environments and through the incorporation of diverse molecular probes. In several CYP systems, control reactions lacking the specific substrate showed comparable or higher peroxide *in milieu*, thereby discrediting the foundations of the erstwhile hypothesis. The profiles obtained by altering CYP:CPR ratios and the profound inhibitions observed upon the incorporation of catalytic amounts of horseradish peroxidase confirm the obligatory roles of DROS *in milieu*, ratifying *murburn* as the operative concept. The mechanism of uncoupling (peroxide/water formation) was found to be dependent on multiple one and two electron equilibriums amongst the reaction components. The investigation explains the evolutionary implications of xenobiotic metabolism, confirms the obligatory role of diffusible reactive species in routine redox metabolism within liver microsomes and establishes that a redox enzyme like CYP enhances reaction rates (achieves catalysis) via a novel (hitherto unknown) modality.

## Introduction

To maintain structural and functional integrity, animals expel the “deleterious and non-constitutional” extraneous molecules that enter their system. Xenobiotics are such a category of molecules and liver, the sentinel organ, deals with them (Testa, 1995). On the microsome membranes of hepatocytes are found copious amounts of diverse isozymes of the heme protein cytochrome P450 (CYP), with much lower distribution densities of a unique flavoprotein reductase (CPR; Guengerich, 2004; Nelson, 2009; Xia et al., 2011). Working in tandem, these two proteins carry out the phase I metabolism of most xenobiotics (Testa, 1995; Guengerich, 2004). The reaction avails redox equivalents from NADPH, with oxygen also serving in the overall mixed-function oxidase scheme. The process may recruit cytochrome *b*_5_ (Cyt. *b*_5_) too. Thus, the tetra- or penta-component system localized on the microsomal membrane attacks xenobiotics, rendering them more polar (e.g.,—by hydroxylation) or breaking them up into smaller molecules (e.g.,—by heteroatom dealkylation).

**Figure 1:**
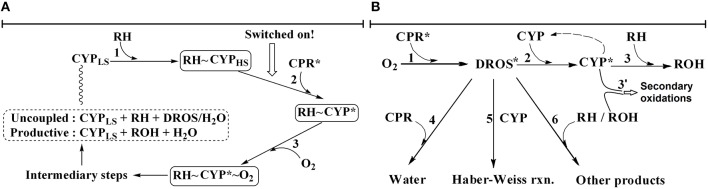
**Schema of old and new hypotheses explaining CYP + CPR mediated xenobiotic metabolism**. The presence of NADPH is implied. RH and ROH stand for substrate and product, respectively. Asterisk is used to indicate an activated species. **(A)** The erstwhile “textbook” CYP + CPR catalytic cycle: The thermodynamic switch is a CPR-based push that is dependent on substrate binding to CYP. All events leading to product and DROS formation require the substrate bound to CYP. **(B)** The newly-proposed *murburn* concept: Substrate is required only in the last step, there are no protein-protein complexations and all reactions are randomized and bimolecular. The electron sink afforded by substrate hydroxylation or ROS depletion serves as a redox pull at right.

The prevailing mechanistic understanding as per textbooks (Testa, 1995; de Montellano, 2015), reviews (Guengerich, 2004; Denisov et al., 2005; Shaik et al., 2005; Poulos, 2014) and reputable publication portals (Rittle and Green, 2010) is captured in the sequence of events depicted in Figure 1A. The first and obligatory step is the binding of xenobiotic substrate at the heme-pocket of the pertinent CYP. Supposedly, this step changes the spin state of heme-Fe and increases the redox potential of CYPs with respect to the CPR. The favorable gradient thus created serves as a “thermodynamic switch or push,” enabling reduced CPR to pump electrons to diverse CYPs via long-distance electron tunneling through the protein-protein complex. Once the heme-iron center is thus reduced, molecular oxygen binds there. The oxygen bound at heme-center gets further activated and forms Compound I, the presumed catalytic intermediate. Thereafter, with an efficient substrate, a facile “oxygen rebound” results in substrate hydroxylation. The product thus formed loses affinity and detaches from the heme-pocket. In the presence of an “inefficient substrate,” diffusible reactive oxygen species (peroxide, superoxide, etc.; deemed as deleterious side-products in this scheme) and water are formed at the heme-center and released subsequently.

It is difficult to envisage that a plethora of xenobiotics (of varying dimensions, topographies and surface electrostatics) could stay “committed to catalysis” (Lu, 1998) by remaining glued to the same heme-pocket for protracted time-frames. For the lack of significant affinity-based causatives, the erstwhile cycle looked too “deterministic” (Venkatachalam et al., 2016). Protein-protein or protein-small molecule complexes/crystals may spontaneously form when water is removed from a system comprising of hydrophobic elements. We argued that there is little evidence for the functional roles of such complexes *in situ* (Venkatachalam et al., 2016). Further, when the enzymes and reactants are taken *in vitro* at nM and μM ranges respectively, we could observe specific product formation at high rates, in the range of ~0.1–1 s^−1^ (pseudo-first order), which is unaccountable by the erstwhile hypothesis' multi-molecular and ordered sequence of events. Based on such evolutionary/chemical logic, diffusion/collision argument, some meta-analyses of kinetics data and *in silico* evidences (Venkatachalam et al., 2016), mechanistic findings on a polar heme-thiolate fungal enzyme chloroperoxidase (CPO; Manoj, 2006; Manoj and Hager, 2008), fundamental revamping of the mechanism of electron transfers in protein-protein and protein-small molecules (Andrew et al., 2011; Gade et al., 2012; Parashar and Manoj, 2012; Parashar et al., 2014b; Manoj et al., 2016a,b) and some preliminary results on the CYP + CPR reaction systems (Manoj et al., 2010a,b; Gideon et al., 2012; Parashar et al., 2014a), we had proposed a “radical” (pun intended!) explanation with the *murburn* concept (Venkatachalam et al., 2016; Manoj et al., 2016b). Key elements of this “constitutive” paradigm are depicted in Figure 1B. Herein, the presence of CPR generates one-electron redox equivalents from NADPH, which is relayed to the CYP (via diffusible radicals like superoxide OR non-specific electron relays can reduce the heme-center via the proximal thiolate port, and molecular oxygen could coordinate at the distal site thereafter), resulting in the stabilization of a one-electron species (like Fe-superoxide), at the heme-center. The xenobiotic substrate, transiently bound (before/after the radical stabilization event) anywhere on the CYP has an enhanced probability to react with the intermediate, within/on/around the CYP. This scheme is unordered and all steps are bimolecular. The current manuscript probes steps 1 through 3 of the erstwhile hypothesis, explores the complexities of the reaction milieu and checks key predictions we made earlier (Venkatachalam et al., 2016), to ratify the *murburn* concept's applicability to CYP + CPR systems.

## Materials and methods

Most details of materials and methods employed in the current study are standard protocols that have been described previously/recently in our manuscripts (Manoj et al., 2010a,b, 2016b; Parashar et al., 2014a; Parashar and Manoj, submitted).

### Materials

CYP3A4 (P2377), CYP2D6 (P2283), CYP2E1 (P2948), and CYP2C9 (P2378) baculosomes were procured from Invitrogen (PanVera) and a CYP2C9 baculosome was from Merck (#011902). The activity of CYP2C9 and CYP2E1 baculosomes agreed with the statement provided by the supplier. The details of composition are available from the manufacturers' website. A pure CPR prep was also procured from Invitrogen (currently, Thermofisher, P2309). CPO was a gift from the late Lowell Hager (UIUC) and SOD, catalase, HRP, myoglobin and hemoglobin were purchased from Sigma. The sources of pure proteins (CYPs and CPR, prepared from cDNAs) are the same as described in our earlier works (Manoj et al., 2010a,b). Chemicals (of AR grade) were from Sigma-Aldrich, Lancaster or Alfa-Aesar.

### General protocols

In order to gain kinetic and stoichiometric insights, the CYP + CPR reaction was approached in two modalities- (i) A simple laboratory reconstituted system comprising of a mixture of purified proteins (CYP and CPR) at known concentrations in phospholipid micelles or vesicles and (ii) The commercially available baculosome system in which CYP and CPR (and when needed, Cyt. *b*_5_) are co-expressed using baculoviral infection of insect cells. Usually, in baculosomes, CPR is in excess of the actual concentrations present in liver microsomes and these preparations are optimized to ensure high catalytic activity of the CYPs. The dynamics of electron transfer in CYP + CPR reaction systems can be studied at four levels- consumption of oxygen, disappearance of NAD(P)H, production of superoxide/peroxide (diffusible reduced oxygen species or DROS) and conversion of the final substrate to product(s). To minimize experimental variables, the reactions were carried out in aerated vials. Since superoxide equilibrates with peroxide and it is experimentally difficult to quantifiably differentiate their absolute concentrations in a mixture, only the latter was assayed. To minimize the wastage of expensive NAD(P)H in routine experimentations, stocks prepared on first day were used within the next few days also. Therefore, the reactions may have contained up to 20% excess NAD(P)^+^, in conjunction with the actual initial value of NAD(P)H quoted.

The concentrations of CYP stocks (commercial sources and membrane fractions) were determined by CO binding spectra (Omura and Sato, 1964). CPR and NADPH were quantified spectrophotometrically at 550 nm (Pritchard et al., 2006) and 340 nm (Stocchi et al., 1985), with molar extinction coefficients of 21000 and 6220, respectively. The absolute concentration of a commercial peroxide stock was determined by titanium complex formation (Sellers, 1980), which is more accurate than the UV spectrophotometric estimation procedure at ~240 nm. The standardized peroxide stock was then used for preparing the standard plot (of micromolar levels of peroxide) and for estimation of unknown concentrations by Peroxoquant method of Pierce chemicals (Jiang et al., 1990). The standard plot was repeated for each set of samples analyzed, since older reagents gave lower slopes. Peroxide concentration assays were very precise and accurate (~5% standard deviation) with respect to samples drawn from the same vial; however, the standard deviations were generally higher when a reaction was duplicated in another vial. Unless otherwise mentioned, all incubations were done in aerated open vials at 37 ± 1°C in 100 mM phosphate buffer, pH 7.4 and reconstituted systems had 10 μg/ml of 0.2 μm vesicles of dilauroyl phosphatidylcholine (DLPC, Avanti Lipids). The details of HPLC analysis is given in our earlier works (Manoj et al., 2010a). The data points obtained with fluorescence analysis of HFC (Kenaan et al., 2010) correlated well with the HPLC method. *In silico* protocols employed herein have been previously described (Venkatachalam et al., 2016). Other specific details and the initial conditions of assays are listed in the pertinent legends. Values reported are means and standard deviations from duplicate or triplicate assays.

### Inclusion of excess redox-active heme-proteins and dyes

At 5 nM (Invitrogen) CYP2C9, 100 μM diclofenac and 125 μM NADPH, 25–125 nM of myoglobin/hemoglobin (Met-Mb/Met-Hb, both of which have a far higher positive value of redox potentials when compared to CYPs) were incorporated. At 15 min incubation, ~1.15 nmoles/ml 4′hydroxydiclofenac (4′OH diclof) and ~2.2 nmoles/ml peroxide were formed in the control reaction, which consumed NADPH at a rate of 0.225 nmoles/ml/min. The test samples gave a slight increase in NADPH consumption (~10%) and Met-Hb reduced the product whereas Met-Mb increased the product (both by less than 5%), and incorporation of both proteins lowered peroxide *in milieu*. Excess of small redox-active dyes of varying single and double electron redox potentials like- positively charged methyl viologen (E^o^′ ~ −446 mV) and phenosafranin (E^o^′ ~ −273 mV) and negatively charged anthraquinone disulfonate (E^o^′ ~ −184 mV) and indigocarmine (E^o^′ ~ −125 mV) -were incorporated into baculosome and reconstituted systems exhibiting comparable hydroxylation efficiencies. Initial concentrations were- 200 μM diclofenac, 1 mM NADPH and redox sensitive dyes were at 2 μM. Reconstituted system and Merck baculosome preparation reactions were carried out at 200 nM (CPR was ~400 nM) and 10 nM, respectively for CYP2C9. The test samples incorporating the redox sensitive small molecules showed only ~10% variations from the control for the hydroxylation of diclofenac by CYP2C9 in both the reaction setups (after 30 min' incubation, 2.8 nmoles/ml for reconstituted setup and 4.9 nmoles/ml for baculosomes).

### Hydroxylation without protein-protein complexation

Stock solutions of 8 μM pure CYP2C9, 3 μM pure CPR, 200 mM Diclof sodium, 100 mM NADPH and 20% v/v glycerol were made in 100 mM potassium phosphate buffer, pH 7.4. The test reaction was conducted in a 2+4 ml mixture. The 2 ml taken in the dialysis tubing (Spectra/Por, 12–14 KD cutoff) had 150 μl of pure CYP2C9, 200 μl of Diclof, 40 μl of NADPH, 300 μl of glycerol-buffer, and 1310 μl of buffer. The 4 ml taken in free solution had 800 μl of pure CPR, 600 μl of Diclof, 120 μl of NADPH, 900 μl of glycerol buffer and 3580 μl of buffer. The positive control (1ml) reaction had 18.8 μl of CYP2C9, 100 μl of CPR, 20 μl of NADPH, 150 μl of glycerol buffer and 611.3 μl of potassium phosphate buffer. The reactions were gently stirred with a magnetic paddle and incubated for 45 min at 37 °C.

### Simulation of CYP-CPR activity with CYP2C9-DROS

Superoxide stock for this experiment was prepared as follows- ~5 mg of KO_2_ was weighed and dissolved in 700 μl of 50:50 dimethyl sulfoxide (DMSO) and 18-crown ether to give a superoxide stock solution of ~100 mM concentration. 2 μl of this solution was added to 300 μl of 50:50 DMSO and 15-crown ether to give a KO_2_ solution of ~667 μM. 10 μl of this sub-stock was added to 400 μl of the reaction. Therefore, the reaction also had ~1% DMSO and 15-crown ether. It is important to note that the actual initial concentration of superoxide may have been much lower than 16 μM, because superoxide readily absorbs moisture (while weighing out and from the DMSO added). The water molecules provide sufficient protons for the formation of peroxide from superoxide (Sawyer and Valentine, 1981).

## Results

### Effect of substrate and various reaction components on peroxide dynamics *in milieu*

**Figure 2:**
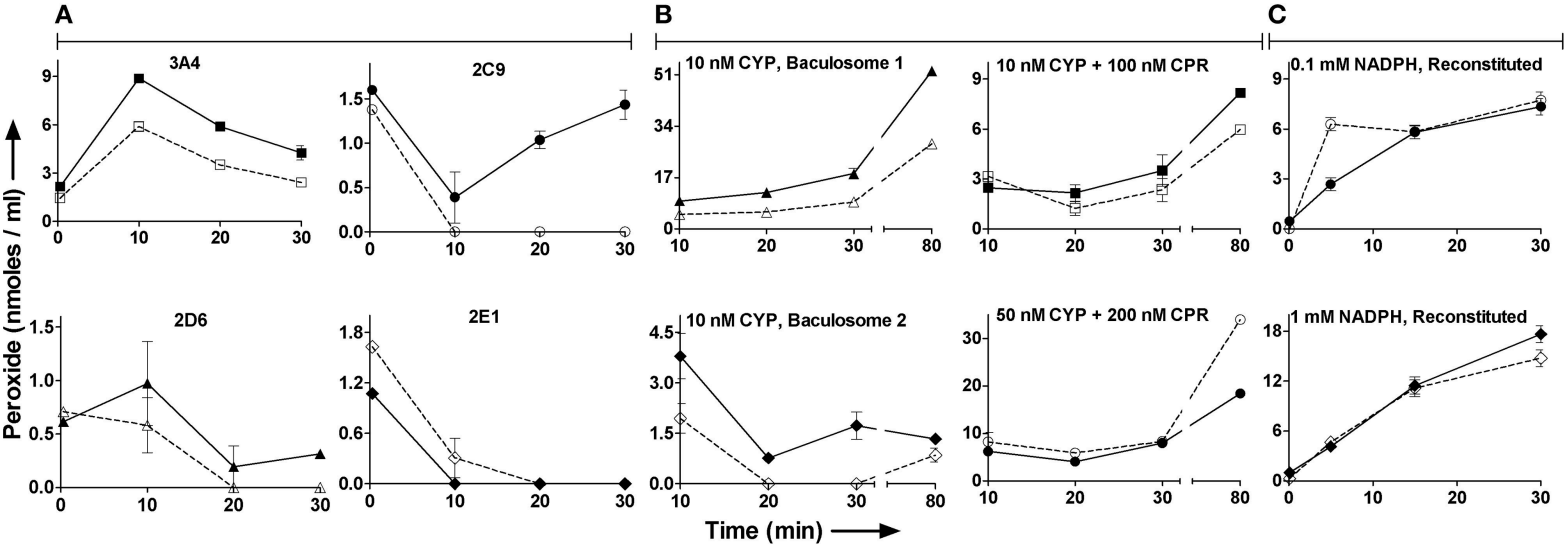
**The effect of substrate on peroxide profile *in milieu* for different CYP reaction setups: Unfilled points are reactions with substrates whereas the filled points lack substrates**. At 15 min incubation, the specific product in CYP2C9 systems ranged from 0.02 to 10 nmoles/ml. **(A)** Peroxide profiles in various CYP baculosomes: Initial conditions- [NADPH] = 2 mM and [CYPs] = 20 nM. Substrates, when present, were at 200 μM. **(B)** Peroxide formation in various reaction setups of CYP2C9: Reactions contained 1 mM NADPH and 200 μM Diclof. **(C)** Effect of NADPH on peroxide profile in reconstituted CYP2C9 systems: Initial conditions- [Diclof.] = 200 μM, [CYP2C9] = 20 nM and [CPR] = 250 nM. At 15 and 30 min incubation, the reaction with 1.0 mM or 0.1 mM NADPH gave 0.14 or 0.13 nmoles/ml and 0.27 or 0.20 nmoles/ml 4′hydroxydiclofenac, respectively.

**Figure 3:**
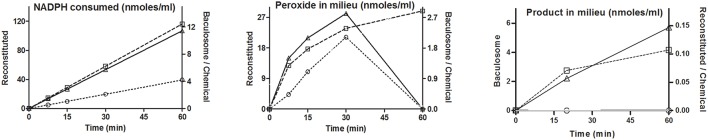
**Temporal profiles for reactants and products in a simple CYP + CPR reaction system: (Key: ○- chemical, □- reconstituted, △- baculosome)**. Initial concentrations of components were- [Diclof.] = 200 μM, [NADPH] = 180 μM. [CYP2C9] = 10 nM. In reconstituted system, CPR was taken at ~0.6 μM.

Figure 2 shows the time course profiles for peroxide *in milieu* for CYP3A4, CYP2C9, CYP2E1, and CYP2D6 baculosomes; with or without their “specific” substrates testosterone, diclofenac, chlorzoxazone, and dextromethorphan, respectively. Most time points showed somewhat lower or similar peroxide concentrations with respect to the pertinent controls lacking the substrate. Also, peroxide concentration lowered after an increase, or varied rather unpredictably over time. Figure 2B shows similar results for peroxide profiles for two sources of baculosomes and varying concentrations of reconstituted pure protein setups of CYP2C9 ± diclofenac. Figure 2C compares the effect of NADPH concentrations in the CYP2C9 reconstituted system. While the initial rates of hydroxylation do not change by increasing NADPH from 0.1 to 1 mM, the peroxide profiles are found to differ. At later time points, lesser hydroxylated product is noted at low concentrations of NADPH in the reconstituted system. Comparatively, baculosomes showed similar profiles for substrate hydroxylation and peroxide formation, in both cases (results not shown). Time profiles and analyses of reaction stoichiometry for two CYP2C9 reaction setups, along with the chemical control, are shown in Figure 3 and Table 1. Linearity could be noted for NADPH depletion for all setups into micromolar ranges, indicating a zeroth order dependence (or consumption via radical route). As the reaction progressed, peroxide:NADPH and product:NADPH ratios lowered whereas water:NADPH ratio increased. In the test reactions, peroxide:product ratio decreased and water:product ratio increased over time. These aspects of the reaction phenomenology clearly establish the inapplicability of the erstwhile hypothesis and indicate multiple redox equilibriums *in milieu*.

**Table 1:** **Comparison of relative stoichiometries of a reconstituted and baculosome system**.

**Rxn**.	**NADPH (nmoles/ml/min)**	**H_2_O_2_/NADPH (%)**	**Prdt./NADPH (%)**	**Water/NADPH (%)**	**H_2_O_2_/Prdt**.	**Water/Prdt**.
Baculosome	0.191 ± 0.01	43^*^, 0	57, 50	0, 50	0.75, 0	0, 1
Reconstituted	1.936 ± 0.05	61, 25	0.2, 0.1	39, 75	339, 278	217, 833
Autocatalysis	0.070 ± 0.02	100^*^, 0	~0, ~0	0, 100	∞, ∞	∞, ∞

The absolute peroxide concentrations in baculosome, reconstituted and autocatalytic systems were (2.1, 0), (17.7, 28.9) and (1.1 and 0) nmoles/ml, respectively at 15 and 60 min. Specific hydroxylated product formed was (1.66, 5.71) and (0.053, 0.11) nmoles/ml for baculosome and reconstituted systems, respectively at 15 and 60 min. Calculation of water formation was: NADPH consumed − (product formed + peroxide formed). Initial conditions are given in the legend to Figure 1. (

* slight overshoot in peroxide estimation is noted).

To understand the dynamics of DROS in the penta-component CYP + CPR reaction system (besides the two key enzymes, NADPH, molecular oxygen and the substrate molecule constitute the five minimal components), the reaction system was studied for peroxide formation, with a lesser number of variables. From Figures S1, S2 (Supplementary Information), it can be noted that peroxide formation dynamics was dependent on the type of substrate present in several types of controls (CPR + Substrate + NADPH, Fe + NADPH + Substrate, Substrate + Superoxide, etc.). The role of substrate serving in modulating the DROS dynamics on its own merit is hereby brought forth. A unidirectional correlation of peroxide formation with progression of time cannot be seen in these cases, quite similar to the complex CYP reaction system. When lesser amounts of ferric citrate was used, the detectable concentration of peroxide went down (along with the NADPH consumption rate; results not shown). From Figure S2 (Supplementary Information), we can infer that addition of excess superoxide (stabilized in DMSO) did not translate into peroxide even within the first few minutes of reaction initiation, indicating that superoxide to water conversion was very efficient under these conditions. Figure S3 (Supplementary Information) probes the ability of various reaction components' roles in depletion of DROS. When 100 μM peroxide was presented initially to various controls, it was seen that all reaction components have the ability to modulate the dynamics of ROS. The setups with CYPs showed the lowest peroxide depletion and NADPH + peroxide showed the highest peroxide dismutation. When we added 10 times more CYP2C9 and brought it to 400 nM, the peroxide concentration was highly stable for a prolonged time (result not shown). This observation indicates that CYPs stabilize the one-electron equivalents generated *in milieu*, which otherwise react with peroxide (or among themselves) to form water. It counters the presumption that CYPs make water at the heme-center. Figure S4 (Supplementary Information) shows that polar ROS scavengers (taken at mM levels) could also deleteriously affect the peroxide depletion activity of CPR (a novel finding we had recently reported; Manoj et al., 2010b). All these observations indicate the presence of intricate redox equilibriums in the reaction system (within both lipid and aqueous microenvironments) and accounts for the chaotic ROS profiles observed in CYP reactions. The findings challenge the prevailing notion that activation of molecular oxygen and generation (or depletion) of ROS primarily occurs at the heme-center.

### Effect of varying the ratio of CYP and CPR on reaction stoichiometry/yield

**Figure 4:**
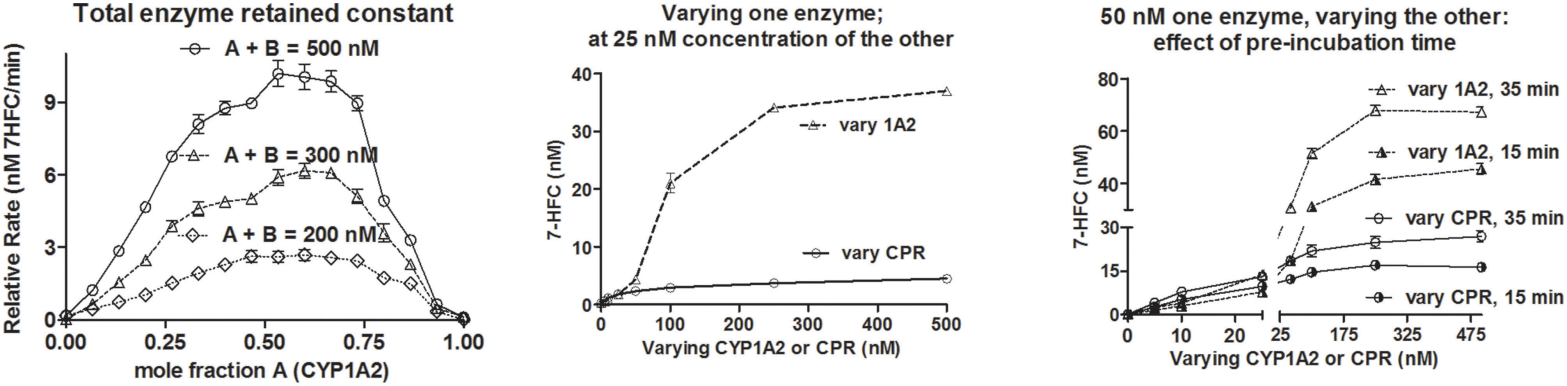
**Searching for functional evidence of CYP-CPR complex formation:** Initial conditions were- 20 μM DLPC, 40 or 50 μM 7EFC, 500 μM NADPH, 0–500 nM CPR or CYP1A2 as titrants. For the left panel, rates were calculated from slope of line obtained by the estimation of product at 3, 6, and 9 min, respectively. For the middle and right panel, reaction sampling was done at 12 and 10 min, respectively.

**Figure 5:**
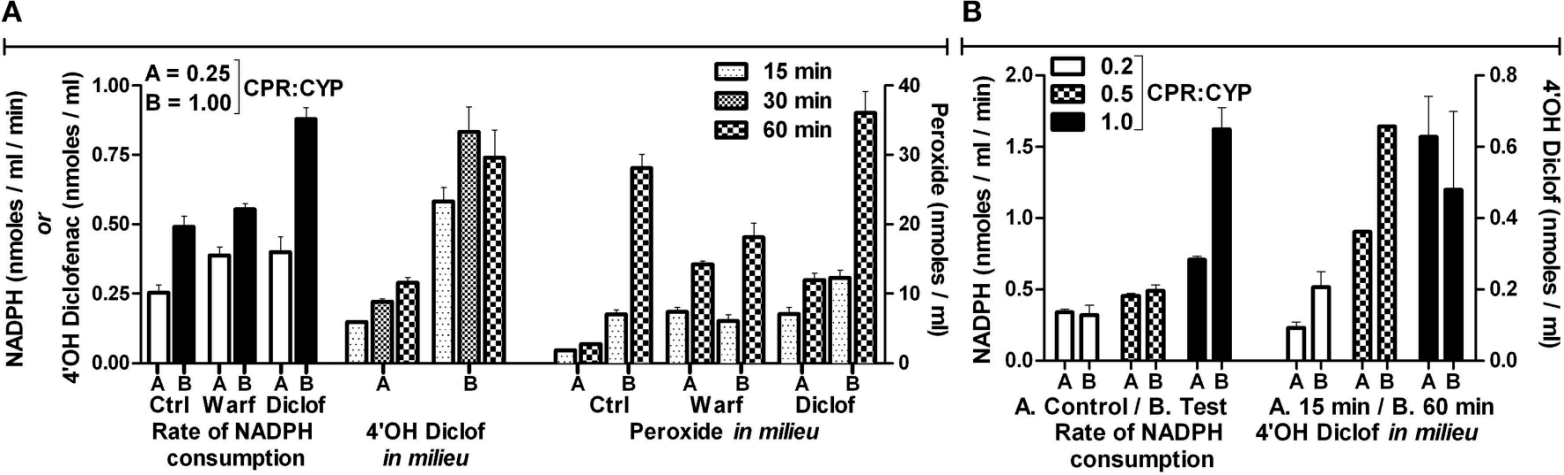
**Monitoring reaction dynamics by varying CPR to CYP ratios in a reconstituted system: (A)** Initial reaction conditions- [CYP2C9] = 100 nM, [NADPH] ~160 μM and [substrates] = 200 μM. Maximal NADPH autocatalytic rates under these conditions were ~0.1 nmoles/ml/min. CYP alone gave ~0.2 and CPR alone gave ~0.3 nmoles/ml/min, respectively. **(B)** Initial reaction conditions were [CYP2C9] = 100 nM, [NADPH] ~200 μM, and [substrates] = 200 μM.

If we were to consider the erstwhile hypothesis as the “binding” principle, increasing CYP or CPR concentration should give more of CYP-CPR collisions and complex formation, which should enhance their functional outcomes in a predictable manner. Figure 4 shows the de-ethylation profiles of a coumarin derivative. When CYP1A2 was varied at a constant CYP + CPR concentration, it resulted in asymmetric curves (left panel). Optimal activities were observed in the range of 0.47 to 0.73 mole fraction of CYP1A2. Varying CYP (at a constant CPR concentration of 25 or 50 nM) gave a sigmoid curve with high amplitude. Whereas, varying CPR (at a constant CYP concentration of 25 or 50 nM) resulted in hyperbolic curve with much lesser amplitude (middle and right panels of Figure 4). Reactions at 25 nM CYP + 100 to 250 nM CPR showed almost an order lesser activity than 25 nM CPR + 100 to 250 nM CYP (middle panel of Figure 4). Overall, the results do not indicate any definite complexation stoichiometry. Furthermore, pre-incubation time showed profound impact on the specific product formation at diverse concentration ranges of both enzymes (right panel of Figure 4). This finding dismisses any suggestions that collisions are “effective” amongst the proteins in the phospholipid microenvironment. The data imply that more CYPs per a given CPR concentration enhance the probability for the former to trap the radical intermediates released by the latter. With more CPR per CYP, the reaction is uncoupled, as the diffusible species end up reacting among them. The effect of varying the CPR to CYP2C9 ratio on the reconstituted reaction system was studied in two independent experiments and the results are presented in Figure 5. NADPH consumption rates and peroxide formation depended on the concentration of CPR. At low CPR:CYP ratios, NADPH consumption/peroxide formation profiles of the reactions incorporating good (diclofenac, Diclof) or poor (warfarin, Warf) substrates did not show major differences and fell in a comparable range, much like the appropriate controls lacking the substrates (Figure 5A). Figure 5B reconfirms the interesting finding seen earlier that the overall yield of hydroxylated product vs. NADPH equivalents consumed was higher for low CPR:CYP2C9 ratios, as was also noted for the CYP1A2 de-ethylation reactions. Coupled with our recent revelations that electron transfer between such redox proteins occurs via non-specific relays (Gade et al., 2012; Manoj et al., 2016a), the profiles obtained herein discredit the erstwhile hypothesis and support the *murburn* concept.

### Effect of incorporation of diverse additives to probe the electron transfer mechanism in reaction milieu

Addition of excess (~5–25-folds) of myoglobin and hemoglobin (mammalian soluble heme proteins offering a better redox potential gradient than CYPs) to the CYP2C9 + CPR mixture did not significantly alter the NADPH consumption or product formation rates. When excess (~10–200-folds) of positively or negatively charged redox-active dyes were incorporated in a reconstituted or baculosome reaction system of CYP2C9, the hydroxylation efficiency was not perturbed (For the two statements above, the details are given in Methods Section Inclusion of Excess Redox-Active Heme-Proteins and Dyes). Also, the CYP + CPR reaction functions effectively in spite of the addition of copious amounts of non-redox sensitive proteins like bovine serum albumin and chick albumin. If protein-protein electron transfer mechanisms were involved, one would expect some inhibition owing to non-specific binding of extraneous proteins on to the reactive proteins' surfaces. These (negative) observations, coupled with the findings from Figures 4, 5, downplay the role of protein-protein complex formations and their purported electrostatic interactions for electron transfers *in milieu*.

**Figure 6:**
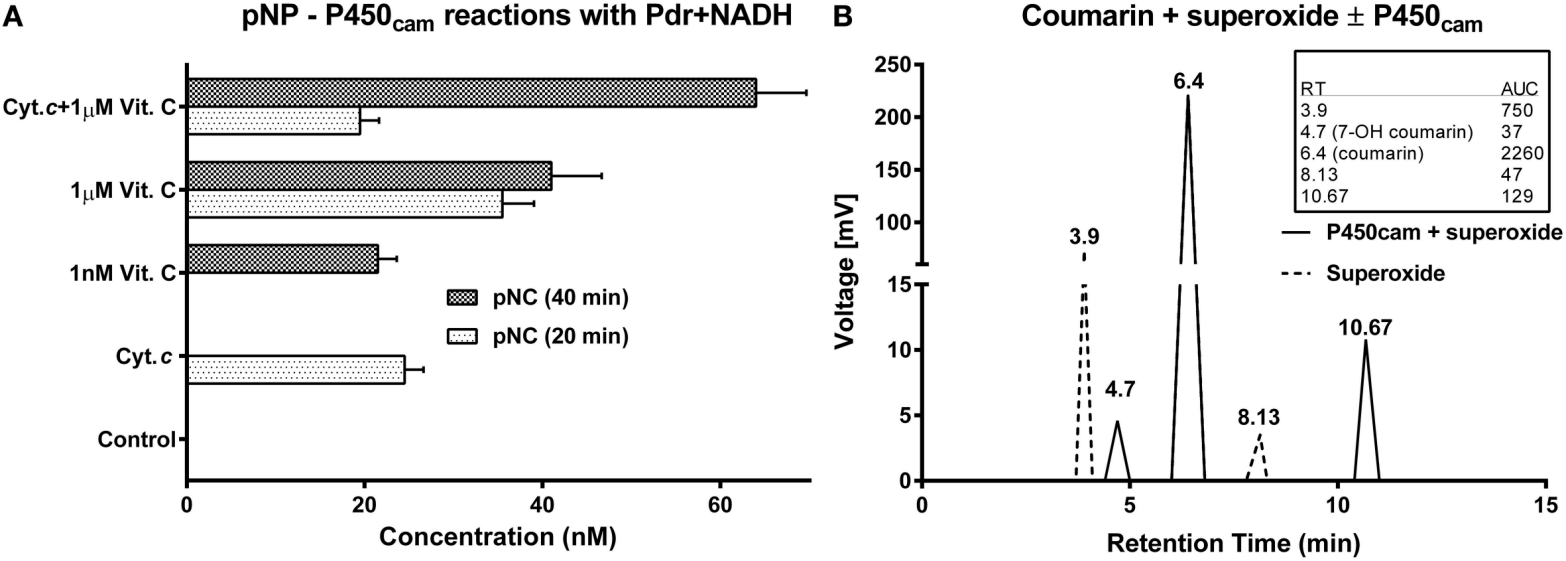
**Demonstration of nonspecific partnering with diverse combinations of P450_cam_, reductases, auxiliary redox partners, substrates and electron donors:** All the reactions were carried out at 27 ± 1°C in 100 mM potassium phosphate buffer (pH 7.4). The initial concentration of substrate employed was 200 μM. Enzyme concentrations were as follows: [P450cam] = 0.5 μM and [Pdr] = 0.6 μM. Electron donors: [NADH] and [H_2_O_2_] = 1 mM, [O${}_{2}^{.-}$] = 50 μM. [Cyt. c] and [Vit. C] = 1 μM. **(A)** P450_cam_ reactions with pNP (a CYP2E1 marker substrate), employing Pdr and NADH as redox equivalent generating agents. The control had only pNP, P450_cam_, Pdr, and NADH. **(B)** Schematic of HPLC profiles obtained with P450_cam_ and coumarin (a CYP2B6 substrate), employing superoxide as the sole redox agent. The inset to the right shows the original area under curve (AUC) values obtained.

**Figure 7:**
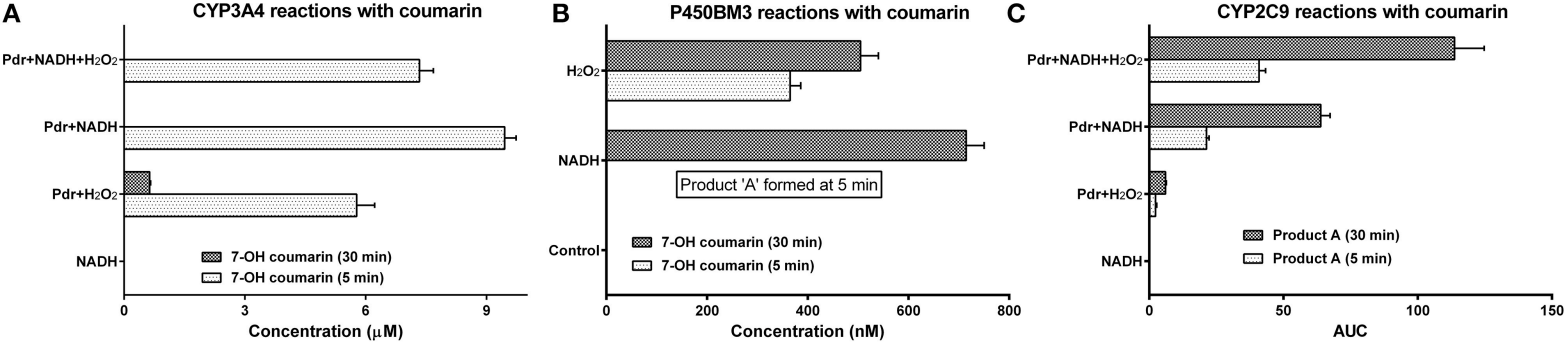
**Demonstration of nonspecific partnering with diverse combinations of CYPs, reductases, auxiliary redox partners, substrates and electron donors:** All the reactions were carried out at 27 ± 1°C in 100 mM potassium phosphate buffer (pH 7.4). The initial concentration of substrate employed was 200 μM. Enzyme concentrations were as follows: [CYP3A4] and [CYP2C9] = 0.1 μM and [Pdr] = 0.6 μM. Electron donors: [NADH] and [H_2_O_2_] = 1 mM, [O${}_{2}^{.-}$] = 50 μM. [Lipid] = 0.5 μg DLPC/pmole P450. **(A)** CYP3A4 reactions with coumarin. **(B)** P450BM3 reactions with coumarin (the control lacked any added reductant). **(C)** CYP2C9 reactions with coumarin.

In Figure S5 (Supplementary Information), it is shown that excess of sulfur-atom containing redox-active small molecules can also affect NADPH consumption, peroxide formation (not shown) and specific product formation (although not in a very profound manner). For example-enhancement of up to 35% NADPH consumption and 15% increase in specific product formation was noted with the incorporation of glutathione. Also, non-linearity (with respect to time) in product formation or NADPH consumption could be noted. We had recently reported the effects of incorporation of two ROS scavenging vitamins in CYP2C9 reactions- the fat-soluble Vitamin E and the water soluble Vitamin C (Parashar et al., 2014a). Figure S6 (Supplementary Information) shows the temporal effects of these vitamins and their derivatives of these vitamins on product formation in two setups- baculosomes and microsomes. We had reported earlier that the water-soluble antioxidant vitamins were less effective, the amphipathic ROS scavengers could effectively inhibit CYP2C9 mediated hydroxylation in both setups, thereby confirming the obligatory role of ROS in the reaction. It can be seen herein that advent of time changes the dynamics (of ROS and thereby, product formation) in reaction milieu. For example- (i) though Vit. C (in microsomes, at 1 mM concentration) inhibits diclofenac hydroxylation significantly at 10 min, the effect wears off at later times. (ii) Vit. E inhibits CYP2C9 baculosome activity completely (in baculosomes, at 0.1 mM concentration) when sampling is done at 10 min, but a significant activity is observed when sample was withdrawn at 15 min. These effects are in line with some of our simulation studies (Figures 6, 7 of the current study and in other reactions with CYP2E1; Parashar and Manoj, submitted ).

The effect of inclusion of DROS utilizing proteins on CYP2C9 mediated hydroxylation of diclofenac is presented in Table 2 and the pertinent chromatograms are shown in Figure S7, Supplementary Information. Inclusion of superoxide dismutase (SOD) and catalase lowered the consumption of NADPH, without significantly affecting the hydroxylations at earlier time points. At later time points, the product yield was higher in reaction mixtures containing catalase and SOD. This is because SOD and catalase lowered secondary oxidations, by removing superoxide and peroxide (respectively) from the aqueous milieu. Chloroperoxidase (CPO) is dysfunctional at pH ≥ 7.0 (where it is ineffective at utilizing DROS) and therefore, it did not significantly affect the reaction profiles in the presence of diclofenac (quite akin to the effects shown by myoglobin and hemoglobin). When CPO was incorporated at pH 6.2 (where it is active) and compared with reactions at pH 7.4, significant reduction of hydroxylation was not seen (Figure S8, Supplementary Information). This could perhaps be because of a glutamate residue located quite adjacent to the heme iron, which could lower the superoxide utilization ability. It was interesting to note that NADPH consumption was higher at pH 6.2 (irrespective of the presence of CPO) but hydroxylations were relatively inefficient. In another experiment (with 200 μM Diclof, 160 μM NADPH, CYP2C9 baculosome −25 nM of CYP), the effect of pH was probed at 3 values- 7.0, 7.4, and 8.0. At an early time frame of 5 min, approximately 12 μM NADPH was consumed and 0.3 μM peroxide was detected in all the three reactions. However, the specific product formation was 2.68, 2.15, and 1.15 μM at pH 7, 7.4 and 8 respectively. At later times, pH 8 showed slightly higher NADPH utilization and significantly greater uncoupling (profile not shown). The observations signify the roles of protons and hydroxyl ions affecting the overall equilibriums.

**Table 2:** **Effect of DROS utilizing proteins on CYP2C9 reactions**.

**Item**	**CYP2C9 only**	**CYP2C9 + CPO**	**CYP2C9 + Cat**	**CYP2C9 + SOD**	**CYP2C9 + HRP**
NADPH rate (- Diclof)	0.35 ± 0.03	1.05 ± 0.02	0.43 ± 0.04	0.39 ± 0.01	4.76 ± 0.07
NADPH rate (+ Diclof)	1.63 ± 0.1	1.77 ± 0.1	0.37 ± 0.08	0.63 ± 0.02	5.97 ± 0.13
4′OH Diclof at 15 min	0.52 ± 0.03	0.54 ± 0.03	0.52 ± 0.04	0.6 ± 0.04	0
4′OH Diclof at 30 min	0.69 ± 0.05	0.71 ± 0.06	0.82 ± 0.07	0.97 ± 0.04	0
4′OH Diclof at 60 min	0.48 ± 0.06	0.40 ± 0.04	1.18 ± 0.09	1.41 ± 0.08	0

Initial concentrations were- [NADPH] = 160 μM, [CYP2C9] ~50 nM, [CPR] ~150 nM, [Diclof] = 200 μM. The proteins procured from commercial sources were added at 0.1–0.5 mg/ml of reaction. NADPH consumption was determined from the slope of linear fit of OD (at 340 nm) at 0.25, 15, 30, 45, and 60 min (except for the reactions containing HRP, for which only the first three points were taken, because all of the NADPH had practically depleted within 30 min). Rate of consumption of NADPH (nmoles/ml/min); 4′OH diclofenac (nmoles/ml).

Remarkably, inclusion of horseradish peroxidase (HRP) increased NADPH by many folds; whereas CYP2C9 mediated diclofenac hydroxylation was completely inhibited (Table 2). In Table 3, the detailed investigation into HRP's intriguing inhibitory effect is shown. The inclusion of HRP took away the peroxide *in milieu*, leading to very high NADPH consumption. HRP is an enzyme that effectively uses both peroxide and superoxide. In conjunction with our findings on the inhibition of CPR-mediated electron transfers by diverse one-electron redox-active agents (Manoj et al., 2016a) and inhibition of soluble heme enzymes' (Parashar et al., 2014b; Manoj et al., 2016b)/CYPs' (Gideon et al., 2012; Parashar et al., 2014a) activities (by various polar and amphipathic DROS- scavengers, respectively), the functional role of diffusible radical species is hereby confirmed in such systems. It can be seen from the controls that the substrate, on its own merit (and in conjunction with the enzymes), can also affect the DROS profiles. This inference was confirmed in another experiment (Supplementary Information, Figure S9) carried out at 27 °C for 1 h, with a commercially available CPR prep, with fully intact N-term trans-membrane peptide. Lower amounts of peroxide generation (<1 μM in all setups) was seen *in milieu*. The obvious inference from both experiments is that both CPR and HRP can utilize NADPH; however, when used in tandem, they yield a hyper-concerted effect, which is significantly augmented by the presence of the substrate. HRP is highly efficient at inhibiting the reaction because it has a high spin Fe (which can flip triplet oxygen to the singlet state!) and it has positively charged amino acids in the active site (His 42, Arg 38 etc., which can better utilize superoxide). Further, it possesses a hydrophobic transmembrane helix on one end (Figure S10 and Table S1, Supplementary Information), which would enable it to compete effectively with CYP. HRP is a fungal enzyme, 325 amino acids long. HRP has only 4% query coverage (BLAST) with respect to the 490 amino acids long CYP2C9. Binding of diclofenac to the enzyme and associated spin change is also inconsequential herein (Supplementary Information, Figure S10). Therefore, CPR-HRP electron transfer by protein-protein complexation (based on a substrate-binding mechanism) can be sidelined. This result provides profound insight into the “redox pull” mechanism operative in the CYP + CPR milieu, quite akin to peroxidases (Manoj et al., 2016b). The findings lend solid support to the *murburn* concept's relevance in CYP catalysis.

**Table 3:** **Effect of HRP on NADPH consumption and peroxide generation**.

***Rxn*. →**	***NADPH only***		***NADPH** + **HRP***		***NADPH** + **CPR***		***NADPH** + **HRP** + **CPR***	
	***−Diclof***	***+Diclof***	***−Diclof***	***+Diclof***	***−Diclof***	***+Diclof***	***−Diclof***	***+Diclof***
NADPH	3.9 ± 0.9	4.2 ± 0.6	12.3 ± 0.9	15.9 ± 1.5	14.4 ± 0.3	15.4 ± 1.4	83.4 ± 2	113 ± 3
Peroxide	0.5 ± 0.2	1.3 ± 0.3	<0.5	0.8 ± 0.3	13.3 ± 1	12.2 ± 0.3	<0.5	1.3 ± 0.2

Diclofenac and NADPH were taken at 200 μM whereas CPR and HRP were at 100 nM each. NADPH-nmoles/ml of the molecules consumed (in 30 min); Peroxide-nmoles/ml formed (at 30 min).

### Simulation of CYP activity with non- conventional redox partners and substrates, sans CPR

We had predicted that if CYPs' activity entailed the involvement of radicals/DROS, it should be facile to simulate the reaction without CYP-CPR complexations, with CYPs and DROS alone (Venkatachalam et al., 2016). Separating the two proteins by a dialysis membrane gave ~1.6% of the specific hydroxylation activity given by the fully mixed system (Supplementary Information, Figure S11, left panel). The lower yield is because in such a scenario, CYP is not present in close enough proximity to latch on to the superoxide or one-electron equivalents unleashed by CPR. Therefore, it is lost to the aqueous milieu, where superoxide dismutates to peroxide (which is a poor activator of CYPs). This inference is confirmed with the fact that it was also possible to efficiently mimic the specific hydroxylation of CYP2C9-diclofenac with stabilized superoxide alone, but not with peroxide (Table 4). This result is quite along the simulation of CYP2E1 activity for pNP substrate (Parashar and Manoj, submitted). Interestingly, when secondary oxidation of diclofenac was probed, it was seen that the original substrate was inefficient at inhibiting the reaction (Supplementary Information, Figure S11, middle and right panels), which negated the “loss of affinity upon hydroxylation” understanding afforded by the erstwhile paradigm.

**Table 4:** **Simulation of hydroxylating activity of CYP2C9 with DROS**.

**Reaction**	**Without CYP2C9**	**With CYP2C9**
CPR+NADPH	Trace	911
H_2_O_2_	<10	~41
O_2_·−	~112^*^	~995^*^

Concentrations of specific 4'hydroxylated diclofenac formed after 10 min incubation (followed by termination of the reaction with the stopping agent) are given in nM. Initial concentrations of the reaction components were- [Diclof] = 25 μM, [DLPC] = 20 μM, [CYP2C9] = 100 nM. When present, the initial concentration of the following components was—[NADPH] = 200 μM, [CPR] = 200 nM, [H_2_O_2_] = 32 μM, [KO_2_] ≤ 16 μM.

* Non-specific hydroxylations or side reactions were noted.

P450_cam_ gave poor query coverage (41%) or maximum identity (26%) with respect to CYP2E1. In Figure 6A, P450_cam_ was used to hydroxylate pNP, a CYP2E1 substrate. In the control, only P450_cam_ and Pdr were used with pNP and NADH; but, this mixture failed to give the product. In the test reactions, Cyt. *c* and vitamin C were employed instead of putidaredoxin (Pdx, the classic redox relay protein in the *Pseudomonas* system). Both redox active molecules effectively supported P450_cam_ mediated hydroxylation of pNP. Further, secondary oxidations were found to be contingent on the type and amount of redox additives present. For example- μM levels of Cyt. *c* gave efficient pNC at initial timeframes but also took away the product at later times. In comparison, nM levels of Cyt. *c* gave pNC only at later time frames, indicating a hastened product formation owing to the change in ROS species in the reaction milieu with the advent of reaction. When coumarin was used as a substrate for P450_cam_ with superoxide, the 7′OH product could be formed, although other side products were also noted (Figure 6B). This is when the reaction of superoxide with coumarin alone did not give significant 7′OH product formation but produced more polar products that eluted upstream (perhaps, owing to multiple hydroxylations).

The results for hydroxylation of coumarin with other CYPs are shown in Figure 7. Coumarin is usually metabolized by CYP2B6 in liver microsomes. CYP3A4 could hydroxylate this substrate using Pdr + NADH or H_2_O_2_ (Figure 7A). Even P450BM3 was able to hydroxylate the substrate to give 7' hydroxycoumarin (Figure 7B). Besides this specific reaction, product A was obtained (possibly hydroxylation at a different carbon, gauging from its elution time in HPLC) in P450BM3 and CYP2C9 reactions (Figure 7C). Quite alike CYP3A4, CYP2C9 also afforded coumarin conversion with Pdr as the redox partner (in conjunction with NADH ± H_2_O_2_). It is highly unlikely that any specific protein-protein complexation modality would be operative in these reaction mixtures, considering the extent of differences in structural and topographical attributes of the proteins/molecules substituted herein. CPR and Cyt. *b*_5_ gave a query coverage of 7 and 37%, respectively and maximum identity of 54 and 25%, respectively (by BLAST analysis) with respect to Pdr and Pdx. These findings also confirm that redox equilibriums evolve and change during a CYP + CPR reaction “in the pot.”

### The effects of reduced nicotinamide nucleotides and cytochrome *b*_5_

Table 5 presents the effect of NAD(P)H on peroxide formation in reconstituted system, with appropriate controls. Peroxide formation increases with time and with NAD(P)H concentration in most of the cases. In the initial time frames, the rate of NAD(P)H consumption and peroxide production increased in the sequence CYP ± diclofencac, CPR ± diclofencac, CYP + CPR, and CYP + CPR + diclofencac. CYP2C9 hydroxylation activities are comparable with high NADH and low NADPH concentrations (results not shown). In these reactions, though NADH consumption was lower than NADPH, the extent of peroxide formation was comparable for 100–200 μM level of reduced nicotinamide nucleotides. This is interesting to note because at least in the initial time frames, the hydroxylation activity is practically zeroth order with respect to NADPH (from a few hundred micromolar to ~ten micromolar concentration).

**Table 5:** **Monitoring peroxide production and reduced nucleotide consumption with controls**.

**Rxn**.	**Peroxide at early time (nmoles/ml)**		**Peroxide at later time (nmoles/ml)**		**Consumption rate (nmoles/ml/min)**	
	**NADPH (10 min)**	**NADH (16 min)**	**NADPH (30 min)**	**NADH (33 min)**	**NADPH**	**NADH**
CYP + Diclof	0	0	0.89 ± 0.2	1.3 ± 0.4	0.13 ± 0.06	0.10 ± 0.01
		3.8 ± 1.9		4.9 ± 0.9		nd
CPR + Diclof	1.96 ± 0.1^*^	1.0 ± 0.1	5.74 ± 0.4^*^	1.8 ± 0.7	0.74 ± 0.03^*^	0.12 ± 0.01
		9.0 ± 0.5		14.2 ± 0.1		nd
CPR + CYP	2.64 ± 0.2	2.9 ± 0.1	6.32 ± 0.1	1.82 ± 0.2	0.90 ± 0.04	0.27 ± 0.03
		12.7 ± 0.7		21.4 ± 0.3		nd
CPR + CYP + Diclof	3.44 ± 0.1^*^	2.1 ± 0.3	6.23 ± 0.1^*^	2.0 ± 0.5	1.08 ± 0.09^*^	0.37 ± 0.03
		11.2 ± 0.5		18.1 ± 1.1		nd

[CPR] = 100 nM, [CYP2C9] = 100 nM and [Diclof] = 100 μM. (The upper row in NADH reactions had 135 μM NADH and the lower one had 1350 μM NADH.) Rate of NADH depletion was determined from the slope of linear fit of 340 nm OD at 0, 90 and 180 min. The controls with CYP or CPR also included Diclof. For NADPH reactions, the initial concentrations of components were–[CYP2C9] = 100 nM, [CPR] = 100 nM, [Diclof] = 200 μM, and [NADPH] ~200 μM. NADPH depletion rate was determined from the slope of linear fit of 340 nm OD at 16, 26 and 36 min. The controls with CYP or CPR did not include Diclof. nd: not determined,

* data previously reported in our work, given here for comparison (Manoj et al., 2010b).

In some preliminary experiments, the specific product formation (yield, efficiency, temporal variations etc.) was studied under different compositions of reaction components (substrate, NADPH, Cyt. *b*_5_, etc.). The results are presented in Figure S12, Supplementary Information. (The reported values therein are good for relative comparison within a given experiment and the absolute values should not be taken for comparison across different experiments). In an experiment with equal amounts of CYP2C9 and CPR (experiment 1, at a relatively lower concentration of diclofenac), it was seen that incorporation of Cyt. *b*_5_ had a “straightening effect” (which is partly interpreted as prevention of secondary oxidations) on the curves. Usually, the addition of Cyt. *b*_5_ had the effect of lowering or enhancing the yield of product, depending upon the reaction conditions (CYP:CPR ratios and CPR, NADPH, and substrate concentrations were crucial determinants). This showed that all these components contributed to multiple redox equilibriums operating *in milieu*. NADPH was a better electron donor than NADH (depending on the reaction compositions, it ranged anywhere from a few folds to an order of magnitude), but a higher concentration of NADPH was correlated to lowered specific product *in milieu* (once again, interpreted to be owing to increased secondary oxidations), particularly with the progression of time. Table 6 shows the comparison of NADPH and NADH as electron sources (at an approximately equimolar starting concentration of both NAD(P)H and substrate), in reconstituted and baculosome systems. The utilization rate of NADPH and NADH were comparable in reconstituted systems and yet, the hydroxylations were less efficient with NADH in all systems. An initial surge or delay in NADPH/NADH utilization was also noted in some reactions. Under these reaction conditions, the incorporation of Cyt. *b*_5_ and DLPC enhanced product formation and lowered NAD(P)H utilization (and thereby, significantly enhanced “coupling” or product yield) in reconstituted setups. In baculosomes, a greater distinction was seen between NADPH and NADH, with respect to reduced nucleotide utilization. This could perhaps be owing to a better interfacial phenomenon in the baculosome systems, where the enzymes are well-housed. At certain instances (in the baculosomes), incorporation of Cyt. *b*_5_ shows an enhanced utilization of reduced nicotinamide nucleotides and product yield (particularly, at later reaction times), indicating an intricate interactive dynamics of DROS evolution and utilization in the system (result not shown). Also, the ability of DLPC to affect diclofenac hydroxylation yield depended on the initial concentration of diclofenac and the extent of breakage of N-term of CPR (result not shown). Figure S13 confirms the findings of Table 6, with another CPR preparation, at comparable concentration terms of the reactants. Here too, in the reconstituted system, incorporation of Cyt. *b*_5_ lowered NADPH consumption (for example- from 15 to 60 min of reaction time, the setup lacking Cyt. *b*_5_ consumed 53 nmoles/ml NADPH whereas the reaction incorporating the Cyt. *b*_5_ consumed 26.5 nmoles/ml). Here, we could note that in the reconstituted setup, the incorporation of Cyt. *b*_5_ was not beneficial in the early time frames for NADH reactions. This signifies that in such a system, sequestering of the one-electron equivalents by Cyt. *b*_5_ was deleterious for the specific product formation.

**Table 6:** **Effect of reaction conditions on the utilization of reduced nicotinamide nucleotides and specific hydroxylated product formation**.

**Reaction setup**	**Sampling at 15 min**				**Sampling at 60 min**			
	**Reductant consumed**		**Product formed**		**Reductant consumed**		**Product formed**	
	**NADPH**	**NADH**	**NADPH**	**NADH**	**NADPH**	**NADH**	**NADPH**	**NADH**
1.2C9 + CPR	44.1	40.9	0.623	0.089	76.9	85.1	2.657	0.319
2.2C9 + CPR + Cyt. *b*_5_	39.6	42.0	0.652	0.093	71.4	86.9	2.913	0.330
3.2C9 + CPR + DLPC	29.5	28.8	0.761	0.107	71.1	72.5	3.522	0.406
4.2C9 + CPR + Cyt. *b*_5_+ DLPC	28.5	25.7	0.930	0.154	70.0	65.2	4.078	0.638
5. Baculosomes	36.9	16.6	3.013	0.254	61.4	19.2	8.313	0.870
6. Baculosomes + Cyt. *b*_5_	33.3	nd	4.522	0.554	63.9	nd	15.61	1.971

Initial concentrations in reconstituted systems-[CYP2C9] = 170 nM, [CPR] = 600 nM, [Cyt. b_5_] = 40 nM, DLPC = 7.6 μg/ml. In baculosomes, CYP2C9 concentration of 10 nM; [NAD(P)H] ~180 μM and [Diclofenac] = 200 μM. Incorporation of Cyt. b_5_, DLPC and both to the CYP + CPR setup enhanced the reactions by 1.09 ± 0.07, 1.77 ± 0.06, and 2.55 ± 0.2-folds in both NADPH and NADH reactions (at 15 min). All concentrations are given in nmoles/ml.

We probed the *in silico* binding of nicotinamide nucleotides with CPR and the results are given in Table 7. We could not find a molecular explanation (based in structure-affinity correlation) for the enhanced efficiency of NADPH (when compared to NADH) as the electron donor (vis a vis CPR) for the generation of specific hydroxylated product. Both show comparable binding energies and in fact, NADH shows better orientation and energy differences between the reduced and oxidized forms (thereby facilitating a dissociation of the oxidized product).

**Table 7:** ***In silico* analysis of binding of nicotinamide nucleotides with CPR**.

**Substrate (log P)** [log D]^*^	**Blind Docking**			**Centered Docking**		
	**Lowest energy (kcal/mol)**	**Distance (Å)**	**Interactions**	**Lowest energy (kcal/mol)**	**Distance (Å)**	**Interactions**
NADPH (−5.93) [−12.2, −12.86]	−2.01	9.4	TRP679 ARG570	−7.33	20.2	GLU482 ARG301
			ARG600 LYS605			ARG570 ARG600
						CYS569 SER599
						LYS605 VAL606
						TYR607 ALA597
NADP+ (−7.3)	−1.34	10.8	ARG570 ARG600	−7.50	11.7	ARG636 THR538
[−8.6, −9.38]						ARG301 ARG570
						ARG600 SER599
						TYR607
NADH (−4.35)	−3.10	9.0	TRP679 ARG570	−7.28	9.3	SER599 ARG600
[−9.1, −9.17]			ARG600 ARG301			ARG570 LYS605
						ARG301 VAL477
						VAL478 TRP679
NAD+ (−5.72)	−2.07	17.1	TRP679 ARG570	−6.02	7.5	ASP641 TYR607
[−5.92, −5.99]			SER680 VAL477			PRO536 ASP634
						THR538 ARG600
						ARG570 ASP575
						ARG301

Distance–NAD(P)H reaction site and FAD reaction site;

* log D values are given for two pH values of 5.5 and 7.4, respectively. Grid B-blind docking; Grid C-heme-centered docking.

**Figure 8:**
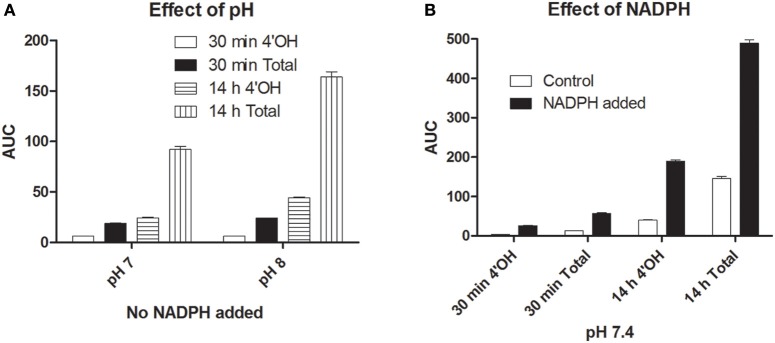
**Hydroxylation of diclofenac by a simple chemical control:** Incorporation of NADPH enhanced rate and specificity. Initial conditions were- [Ferric citrate] = 1 μM, [H_2_O_2_] = 400 μM, [diclof] = 200 μM, [NADPH] = 200 μM, ~OH Diclof area = 45/μM. **(A)** Effect of pH (without NADPH). **(B)** Effect of NADPH (at pH 7.4).

Simple chemical controls employing a high redox potential insoluble Fe (III) species (at the physiological pH 7.4) gave small amounts of hydroxylation of diclofenac, a CYP2C9 substrate. The results are shown in Figure 8. Increasing the pH from 7 to 8 gave a slight increase in hydroxylation efficiency (contrary to the enzyme activity). At pH 7.4, at initial time frames, the control reaction (without NADPH) had ~31% of the specific 4′OH diclofenac whereas the test reaction (incorporating NADPH) had ~46% of the specific product. The presence of NADPH increased reaction rates for the specific 4′OH product by a factor of 6.5 and the total hydroxylation rates were enhanced by a factor of 4.4. Another set of reactions were done employing 1 μM FeSO_4_/dil. acid (where more ferrous ions could be better stabilized), at 1 mM NADPH, 200 μM H_2_O_2_ and 100 μM Diclof. Results for this (not shown) also revealed that NADPH + Fe + H_2_O_2_ combination showed greater hydroxylation rates and specificity (when compared to Fe + H_2_O_2_) for 4′OH diclofenac formation for short/longer incubations. Therefore, these simple chemical controls showed that hydronium and hydroxyl ions from the reaction milieu and reduced nicotinamide nucleotides could also play significant roles in instilling specificity and enhancing rates of hydroxylation (quite akin to the dual function of chloride ion in chloroperoxidase chlorination milieu; Manoj, 2006).

## Discussion

The outcomes of this work indicate multiple one and two electron redox equilibriums in the milieu involving NADPH, substrate, DROS, enzymes, etc.; quite akin to other heme-enzyme paradigms that we recently established (Manoj, 2006; Manoj et al., 2010a, 2016b). Now, we attempt to provide explanations to the overall phenomenology of CYP reactions. Before reading any further, it is strongly advised that the reader familiarizes with the critical dissection of the erstwhile hypothesis and the original proposal we made on *murburn* concept in Venkatachalam et al. (2016).

### Dynamics of ROS *in milieu*

The dynamics of peroxide *in milieu* can be better explained by the oxygen activation and peroxide depletion ability of CPR (Manoj et al., 2010b), the interactive dynamics of superoxide + peroxide *in milieu* and from the results of controls investigated in the current study. The water formation at heme-center by Compound I (Grinkova et al., 2013) remains a highly improbable and unproved proposal. A highly reactive electron-deficient intermediate need not wait indefinitely for two protons and two electrons in the highly hydrophobic lipid layer, in order to form water. It is clearly established that a CYP like 2C9 has limited peroxide utilization ability and it serves primarily as a one-electron species (like superoxide) stabilizer. The controls employed in this study clearly show that the concentration and temporal variation of peroxide formation *in milieu* is modulated by diverse factors and is contingent upon the type of substrate present, even without CYP's presence. Even though a high amount of superoxide was introduced into aqueous phase, within the first minute of the reaction, the majority of ROS were converted to water, and only a small fraction of peroxide was observed. Also, since peroxide levels were higher in substrate + superoxide combinations, it implies that substrate interaction or reaction with reactive species could also facilitate peroxide generation. A high spin iron center could easily flip the triplet oxygen to singlet state, which could react fast with the flavin of CPR to give peroxide. This could afford an explanation for the increased consumption of NADPH (and elevation of peroxide levels) upon the incorporation of CYP into the CPR + NADPH reaction. The involvement of hydroxyl radical or singlet oxygen cannot be ruled out in an aqueous system containing metal/flavin centres, triplet oxygen, superoxide and peroxide. From the data obtained herein, the semiquinone state of the flavin seems to be crucially involved in CPR mediated catalysis. The temporal variations in ROS, products etc. are thus well-accounted by the *murburn* concept. The erstwhile hypothesis' iron-center based ROS production fails to account for the ROS dynamics observed. Higher concentration of CPR would give greater DROS, which would react amongst themselves and therefore, a low concentration of CYP cannot compete effectively for stabilization of the radical species. HRP can effectively utilize the DROS at the phospholipid interface whereas SOD removes the superoxide only from the free aqueous phase and cannot compete with the CYP at the phospholipid interface. The utilization of DROS to hydroxylate the substrate or dissipation to water formation serves as an electron sink, which leads to a redox pull, which in turn releases more electrons from NADPH (via CPR).

### Roles of nicotinamide nucleotides

The most essential aspect of the *murburn* concept is that CYPs' crucial role lies in stabilizing the one-electron equivalents spewed by CPR within the phospholipid bilayer and maintaining “a one-electron, radical reaction paradigm.” Clearly, the zeroth order depletion of NADPH in CYP reactions (Manoj et al., 2010b; and this work) and the ability of NADPH to enhance rate/specificity of product formation in chemical controls signifies a diffusible radical reaction. The second electron is readily available from NADPH, or other radicals/peroxide *in milieu*.

The experimental evidence shows a comparable depletion rate for both NADPH and NADH, at least in some systems. Also, the *in silico* exploration gave no evidence for the enhanced effectiveness of NADPH for the hydroxylation activity. The answer to this conundrum must lie in the physical nature of NADPH/NADP^+^ redox couple (and their partitioning *per se*), and not in its interactive ability with CPR. From the theoretical considerations, a few inter-related aspects are noted that favor NADPH-

1. Redox potentials: NADPH couple has ~5 mV lower two-electron redox potential than NADH couple and this value can go up in the physiological milieu owing to distribution of the nucleotides in the cell.
2. Partitioning effects: NADPH has ~3 units of log D (at pH 7.4) and ~1.5 units of log P lower than NADH.
3. Catalysis by protons: The difference between the pK_a_ of the two molecules- i.e., the difference of log D at pH 5.5 and pH 7.4 of NADPH is 0.6 units whereas that of NADH is only 0.06 units.
4. The phosphate group of NADPH (or an intermediate/product thereof) may be involved in charge stabilization of the reactive intermediate (which is involved in the final product formation from substrate). Further, the phosphate group of NADPH could aid proton delivery in a crucial step that occurs in free milieu (or interface).

Our observations show that the nicotinamide nucleotides affect DROS profiles and secondary oxidations, which can be essentially understood to occur in the aqueous phase. This supposition is also strengthened by control reactions where addition of NADPH lowers side reactions of superoxide and from the observations that excesses of NADPH is deleterious to the yield of product (results not shown). Further, it is also known that NADPH addition in bulk or production via a regenerating system affects ROS/Cyt. *b*_5_ dynamics and product hydroxylation efficiencies (Porter, 2002; Schenkman and Jansson, 2003; Kumar et al., 2005). Since CPR, CYP and hydrophobic substrates are generally localized in the microsomal membrane, we can safely surmise that the first electron transfer and substrate activation step must occur at the phospholipid interface. We can now infer that the nicotinamide nucleotides could be involved in at least two discrete one-electron steps. Therefore, NAD(P)H plays multiple roles by virtue of its interaction with CPR, ROS and diffusible substrate-centered radicals.

### Protein distribution and interactions

The erstwhile hypothesis involves protein-protein interaction and long-range electron transfers, which is still a gray area in biochemistry (Gray and Winkler, 2003; Moser et al., 2008). The scheme for the same involves collision, conformational gating and subsequently, the actual electron-transfer process. The time required for the movement of electrons across a maximal distance of 12 ± 2 Angstroms (approximately 20 bonds) is in the range of milliseconds. The process gets inefficient with increasing distances too. Such a process cannot be efficient between bulky proteins in the highly constrained and low-energy microcosm of phospholipid membrane. This would require a high concentration of CPR for effective catalysis; also, we would expect better catalytic turnovers and product yields with a high CPR:CYP ratio. However, our works show quite a contrary effect. The well-known poor distribution density of CPR (1 CPR to ~100 CYPs) within the liver microsomes and the promiscuity of CPR (a unique CPR can transfer electrons to hundreds of CYPs) can now be efficiently explained with the *murburn* concept. A higher CPR:CYP ratio would not be evolutionarily desirable, since it may lead to a high rate of DROS generation, depletion of redox equivalents and cause too many secondary oxidations. Therefore, the presence of CPR at low concentrations is an evolutionary requisite. If the substrate does not leach out from the membrane after one-step oxidation and continues to persist near the lipid bilayer, secondary oxidations (in due time, even with lower CPR concentrations) would ensure that it would become more polar, thus causing its removal from the vicinity of the membrane. The error bars are bigger for reconstituted systems with higher CPR (particularly at later time frames) because of the greater amounts of chaotic ROS involved. The very notion that processes featuring a diffusible intermediate are always chaotic is misplaced. One of us has shown in CPO's chlorinating mixture that at a lower concentration of a diffusible reactive agent, one can get fairly reproducible and non-chaotic specificity (Manoj, 2006). Subsequently, we have established that electron transfers by diffusible agents are highly reproducible (Manoj et al., 2016a). Further, the same idea has been demonstrated in a simple chemical control for diclofenac hydroxylation in this work (Figure 8).

It is noteworthy that till date, there exists no direct experimental evidence or justification regarding the necessity of CYP-CPR complexations for the overall catalysis in a dynamic state. The existence/elucidation of various homo- or hetero-oligomers in CYP-CPR mixtures is not questioned, but the obligatory relevance of the same in the overall catalysis appears to be doubtful. The deleterious effect of higher ionic strengths or mutations of key amino acids on the overall reaction (Voznesensky and Schenkman, 1994; Shen and Kasper, 1995) cannot be interpreted to be because of a disruption of electrostatic interactions between CYP and CPR alone. In the work by Shen et al. (Shen and Kasper, 1995), the electron transfer rates increased sharply when ionic strength was increased at lower concentration ranges. We have confirmed this with experiments on Cyt. *c* reduction by intact CPR. Since pH is the primary determinant of the charged states of the amino acids touted to play roles in CYP-CPR interactions, enhancement of activity at lower ionic strengths does not go well with a protein-protein complexation process. It goes perfectly well with the concept that diffusible species (including ions) mediate electronic relay in these heterogeneous systems. Mutating residues located far away from the active site (even amino acids located on the surface of the protein) significantly affected the catalytic rates in a heme-thiolate protein (which did not require protein-protein complexation) like chloroperoxidase (Rai et al., 2000). The ability of various amino residues' (located far away from the heme-center) to modulate ROS (or the “flow” of such species thereof) have been discussed earlier in our works (Gideon et al., 2012; Venkatachalam et al., 2016). So, the effects attributed to a disruption of putative protein-protein interaction by mutation studies can be explained by other considerations also. The data presented in Figures 4–7 and Tables 2–3 argue against the roles of binary complexes of CYP and CPR in routine *in vitro* or *in vivo* scenarios. There are several examples for such non-specific interactions in the literature (Granvil et al., 2002; Lu et al., 2003; Oshima et al., 2004; Kumar et al., 2005). While the *murburn* concept poses no conflict with such observations, application of the erstwhile hypothesis for explaining such data mocks Occam's razor. The different proteins/molecules substituted *in lieu of* the original protein have little structural homology with the latter. Diffusible species (like superoxide) can be a source of activation, leading to CYP-specific product formation, whereas the dismutated product (peroxide) is less effective. This inference is supported by our own observations, in control experiments where peroxide concentration *in milieu* was monitored after addition of superoxide to the solution containing only the substrate (Results Section and Supplementary Information, Figure S2). When CYP molecules are absent from the vicinity of CPR and when the latter releases superoxide, one- electron equivalents, the lifetime of the reactive species is very low because the components of milieu may consume them or they may react amongst themselves. A similar phenomenon is seen in CPO's chlorinating mixture where the diffusible reactive intermediate is taken up by the reaction components themselves, in the absence of a suitable substrate (Manoj and Hager, 2001, 2008; Manoj, 2006). Therefore, it can now be understood that the same CPR can promiscuously “reduce” (or transfer electrons) to a hundred different CYPs, cytochromes and other small molecules only because the interaction is mediated via diffusible species.

### Roles of phospholipid bilayer and cytochrome *b*_5_

Clearly, the effects of partitioning and distribution of ROS and radical scavengers are evident in the reaction systems. The hitherto held belief was that Cyt. *b*_5_ serves as a direct electron donor to the CYPs in certain cases and in some others, it serves as a conformational modulator of CYPs (Porter, 2002; Schenkman and Jansson, 2003; Guengerich, 2005; Kumar et al., 2005). Both solicited protein-protein complexation as the modalities for this electron transfer. We have observed that Cyt. *b*_5_ either enhances, lowers or minimally perturbs the hydroxylation efficiency in the CYP2C9 (Table 6 and Supplementary Information Figures S12, S13) and CYP2E1 (Manoj et al., 2010a; Gideon et al., 2012) systems, depending upon slight or major variations in the overall reaction components' composition. This is backed by literature (Kumar et al., 2005). Changing the reductant from regenerating NADPH system to providing an initial high concentration of NADPH also gives different effects (Manoj et al., 2010a). The erstwhile hypothesis cannot account for the lowering of hydroxylation efficiency with increasing Cyt. *b*_5_ because if the latter served as a protein-protein electron transfer shuttler (which is a rate-limiting step in the erstwhile hypothesis), the hydroxylation efficiency should only be increased by increasing the Cyt. *b*_5_ concentration.

CYP, CPR, and Cyt. *b*_5_ are membrane proteins whereas proteins, quite unlike the hydrophilic Cyt. *c* and NADPH. Partitioning of components is introduced by DLPC and it plays a crucial role in reaction outcomes. Increasing or decreasing concentrations of any species brings in discontinuity in effects and heterogeneity in distribution (as exemplified by aggregation and micellization at one hand and enhanced stability of some radicals at low concentrations in selective niches, on the other hand). Our recent work solved the role of the N-term transmembrane segment of CPR in CYP reactions, explaining that the detached transmembrane N-term segment has DROS modulating abilities (Gideon et al., 2012). We have recently demonstrated that redox additives could enhance electron transfers in heme-enzyme mediated reactions by serving as non-specific agents for redox relay (Gade et al., 2012). Therefore, it is opportune to see diffusible radicals in heme-enzyme systems as functionally relevant agents that bring in both chaos (at high concentrations) and order (at lower concentrations). Cyt. *c* and Vit. C can affect P450 mediated catalysis, as shown in Figure 6 and Figure S6 (supplementary Information). It is under this light that the role of Cyt. *b*_5_ can be better understood. One-electron equivalents generated by CPR are transiently stabilized by Cyt. *b*_5_, which in turn could shift the CPR mediated equilibrium (oxygen–superoxide) to the right, to some extent. Further, Cyt. *b*_5_ could serve as a “dynamic storage port,” accepting an electron from superoxide (at high superoxide concentrations), retaining the one-electron equivalents in the lipid phase and releasing the electron to an oxygen molecule (at low superoxide concentrations). This is quite probable, because the redox potential of Cyt. *b*_5_ is much lower (approximately +25 mV; Porter, 2002; Schenkman and Jansson, 2003) than that of Cyt. *c* (+260 mV). But it is inefficient at giving the electron to CYPs because of their relatively lower redox potentials (Margalit and Schejter, 1973) and poorer mobilities of both proteins. The crux of the issue is the availability of superoxide to CYPs within the kinetically favorable time frame. If the rate of release of electron is commensurate with the rate of CYP mediated hydroxylation, we would observe a positive effect on CYP hydroxylations. On the other hand, if excess Cyt. *b*_5_ sequesters the electrons amongst its own kind (or catalyzes futile reactions) and if the reaction mediated by CYPs occur faster than oxygen to superoxide conversion (brought about by Cyt. *b*_5_), then CYP hydroxylations would be lowered. This analysis could explain how the inclusion of Cyt. *b*_5_ lowers peroxide formation in many reactions. Occam's razor does not favor the interpretation that Cyt. *b*_5_ brings about subtle changes in the conformations of certain CYPs (and that too, in a manner dependent on concentration!). Therefore, the effect of Cyt. *b*_5_ is contingent upon the type of CYP and its concentration, the amount of CPR, the lipid, substrate concentration, etc. We have already shown that substrate sponsored inhibitions can be affected by Cyt. *b*_5_ (Figure S12, Supplementary Information and Manoj et al., 2010a). Increasing lipid concentrations only lowers the electron transfer rates from CPR to Cyt. *c* (Manoj et al., 2016a). This means that the effective DROS concentration (for interacting with Cyt. *c*) is lowered and the DROS produced may not find Cyt. *c* in its immediate vicinity. Therefore, it is clear that the phospholipid bilayer serves to bring CYP and CPR in proximity to each other (and their hydrophobic transmembrane segments aid in achieving this mandate; Gideon et al., 2012) and keep the small amounts of DROS restricted around the membrane. We can now deduce that a CYP like 2E1 has a more fastidious requirement for Cyt. *b*_5_ because it does not have large channels to the heme-center and it depends on Cyt. *b*_5_ to keep the one-electron equivalent “credited” within the protein systems, thereby not leading to peroxide or water formation.

### But what about compound I?

Very importantly, the work leads us to infer that the two-electron deficient Compound I cannot be the preponderant active species for CYP catalysis. Compound I is known to be spontaneously formed in peroxidases [that have acid-base or polar catalytic residues (with dissociable protons) in the distal pocket], at very high concentrations of the enzyme and peroxide, with heterolytic scission of the peroxide. Formation of a Compound I in microsomal CYPs via homolytic scission of oxygen-oxygen bond is not demonstrated yet. The claim made by Green's group (Rittle and Green, 2010) does not stand good for microsomal CYPs because CYP119 has a highly polar distal pocket with three threonine residues. Moreover, the generation of the two-electron deficient intermediate was done with a peroxyacid, which would make facile the heterolytic scission.

A high potential intermediate generation cannot be achieved by the cellular system in such a manner. The “remarkable” findings in our group's work (Manoj et al., 2016b) is conclusive evidence for the statement that a one-electron process is the more favorable thermodynamic route, particularly given the low mobility phospholipid environment. (P450BM3 reactions are not to being considered in the discussion here because it is a very different enzyme!) We have clearly demonstrated that at low concentrations of heme-enzyme (in a few nM to tens of nM ranges) and peroxide (in a few hundreds of μM ranges), there is little probability of the peroxide accessing the active site to form Compound I in the most well-known of peroxidases. As we increase the size of hydroperoxide, the catalatic rate decreases in CPO. This result showed the size/diffusion limitations induced by an active site process (Manoj and Hager, 2001). Since CPO has a relatively easily accessible and larger active site (and the reaction pH is more acidic), it depletes hydroperoxides better than HRP through the two-electron Compound I route. (It is strongly advised that the reader goes through the pertinent results and discussion of these earlier papers from our group). CYPs do not have a highly polar distal pocket and as a result, they stabilize a DROS like superoxide (and consequently, peroxide). This inference is amply supported by the pioneering works of Blumenthal and Kassner (1979).

Martin Newcomb was probably right earlier when he doubted the involvement of Compound I as the sole oxidant (Newcomb et al., 2000, 2003). Here are some more reasons why [other than the ones quoted earlier herein and elsewhere (Venkatachalam et al., 2016)]-

1. Till date, there is no evidence that Compound I is relevant in routine assay or physiological reaction conditions for CYPs (particularly, for the hydroxylation of non-activated carbons, which the CYPs are famous for!).
2. There is no peroxidase dismutation activity observed in major CYPs, like CYP2C9 (Manoj et al., 2010b), which is a simple feature expected of Compound I species, well-demonstrated in heme peroxidases. (Surely, peroxide being small, should act as an efficient substrate for CYPs, which can act on such diverse and relatively lesser reactive molecules!).
3. Heme-peroxidases (demonstrated to have Compound I, like CPO) show oxygen insertions of activated carbons only, and give poor efficiency for hydroxylations of non-activated carbons. This shows that Compound I may not have the potential to hydroxylate non-activated carbons.
4. Size and orientation of substrate are not a major constraint to most CYP mediated oxygen insertions but it is an issue with a Compound I species of heme-thiolate enzyme like CPO (Lakner et al., 1997).
5. CYPs swing both ways- carry out oxidations and reductions. This shows a one-electron species role. It is highly unlikely that a two-electron deficient electrophilic Compound I could mediate reductive reaction cycles.
6. Optimized enzyme reactions give microsomal CYP activities with pseudo-first order rates of substrate conversion at 1 per second, when nM levels of CYPs are used in the reaction. If we assume that collisions are at least one order slower in the lipid phase, we are left with a reaction rate that is 10 times higher than the maximum achievable collision rates achievable by such low CYP concentrations. When we consider that time would be taken for the large substrates to diffuse into the heme pocket (for which the F and G loops open in millisecond time frames) and electron transfer across distances of >10 Å become less probable (and which too require greater than millisecond time frames), we cannot explain the reaction outcomes with multiple molecules' repeated affinity-based complexation process.

Therefore, there are very strong points to argue that a Compound I species formation via homolytic scission with NADPH + CPR + O_2_ combine (going through the erstwhile mechanistic route, as shown in Figure 1A) is highly improbable. We speculate (forthrightly!) that it is also highly unlikely that CYPs could form Compound I, even with ROS species like peroxide or superoxide as starting material, particularly with nM levels of CYPs. To us, the probability of small molecule access to heme-center appears kinetically challenged in low enzyme concentrations (Parashar et al., 2014b) in the phospholipid microenvironment and the oxidase (Fenton-like) radical pathway seems more probable. (Disclaimer- The scenario may be different in highly concentrated synthetic chemistry setups and spectroscopy sample preparations!).

When it is known that a CYP can kinetically differentiate between an R and S enantiomer of a substrate (Oguri et al., 1994; Kaminsky and Zhang, 1997), it is highly unusual that most of the human liver microsomal hydroxylations are not enantioselective. (There are exceptions like benzylic hydroxylation of Bufuralol-CYP2D6, which can be explained by considering that the heme-iron active site in such an enzyme is easily accessible through a canal and the locus of hydroxylation on the substrate molecule is not geometrically/sterically hindered. Further, the relevant carbon has higher electron density, or is activated. It is interesting to note that even in such cases, the reaction is not regiospecific, as significant aromatic hydroxylation is also noted). This is quite unlike CPO catalyzed epoxidations of benzylic centers where high enantioselectivity is coupled with high yield (Allain et al., 1993). If one considers that the heme-floor forms the ground for all CYP reactions, it is practically impossible to imagine why a substrate should bind enantioselectively but not react in the same manner (if binding is a relevant and required facet, as the erstwhile hypothesis seeks). It is impossible to imagine how some occluded loci of a substrate molecule can be accessed by a heme-oxygen reactive intermediate. Hitherto, researchers reconciled with the lack of enantioselectivity observed with large substrate molecules and the small intramolecular KIEs in similar substrate molecules (of comparable dimensions). [The latter was attributed to masking! This means that the substrate is not free to rotate in the active site and is relatively restrained in one way. If it is restrained, then there is binding (which is what the erstwhile hypothesis seeks) in a particular orientation and this should give enantioselectivity! We seem to run into incongruities with the erstwhile hypothesis every time!] Enantio- specificity/selectivity (in terms of kinetic preference of a substrate enantiomer OR differentiation of enantiopic faces of the substrate leading to enantiomeric excess of a product) can be afforded even if the substrate bound and reacted at a locus distinct from the heme-Fe center. (However, highly enantioselective reaction product formation is definitely a strong case for heme-centered Fe-O species. This can be achieved for efficient CYPs like P450BM3 and other CYPs with non-occluded channels with suitable small molecules, and that too, only at high concentrations of enzymes and substrates).

Therefore, to keep things simple- the hunt for the protagonist(s) in the context would lead us to three candidates- two-electron (Fe-peroxyl), one-electron (Fe-superoxyl) or uncharged radical (Fe- hydroxyl) species of oxygen stabilized at the heme-center. When there is little enantioselectivity and when the enzyme/reactants are in a diluted state (as most lab assays or physiological conditions are), a diffusible superoxyl/hydroxyl radical species serve as good candidates to explain for all observations hitherto available on CYPs. These have the potentials and these can gain access to remote regions of the substrate molecule. In reactions where LFE correlations give a positive slope with increasing sigma values (that is- the reaction is inversely dependent on the electron density on the substrate or the transition state is negatively charged), superoxide species based reaction scheme seems more probable. Most importantly, such species work in conjunction with NADPH or even two one-electron species could be involved in two distinct steps (since the radical reactions need to be quenched, finally!). A cationic species can also be involved, depending upon how the reactive intermediate forms a stable product. If this species' interaction is rate limiting, we could get its signature also in the overall scheme. Highly enantioselective reactions (with high yields) would most probably have the Fe-peroxyl species reacting with activated centers on the substrate (within the distal active site) or the diffusible radical species reacting with a tightly bound substrate (anywhere on or around the enzyme).

### Cytochrome P450s: what are the issues with the past and what should be unlearned for the future?

A question that Fred Guengerich had asked a decade back (Guengerich, 2004) should be unabashedly rephrased as above. It is not a worthwhile endeavor to bank on the absolute values of classical kinetics/equilibrium constants (*k*_cat_, K_M_, K_i_, K_ss_ or K_is_ or the likes) for CYPs. They mean very little because they do not hold the erstwhile theoretical relevance and they may vary by orders of magnitude, based on the initial and evolving reaction conditions. (When a molecule serves as an activator and inhibitor, and that depending upon concentration, to the same given enzyme; and when this phenomenon is seen too often, with not just a unique additive or substrate, it is high time to move beyond the Michaelis-Menten paradigm to interpret such effects). It is not a good idea to debate over the “rate limiting step” or “a unique catalytic species” in CYP catalytic cycle anymore. There could be any number of processes and molecules that would be operative, which in turn, can easily be envisioned to be dependent on initial assay conditions. It would be a good idea to start getting more statistical information from clinical or *in vivo* research or at least, we should employ microsome preps for getting a more reliable picture of what could really happen. (We should not use baculosomes or reconstituted systems to pinpoint and portray the dynamics within the liver microsomes). The results from *in vitro* should not be used with high confidence to project the outcomes *in vivo* or *in situ*. This is because the presence of a trace amount of a small molecule can alter the whole kinetics, as we have shown with the reductionist approach. It is feasible to predict what position a substrate might get hydroxylated at, but more important is the interaction of intermediates formed, rate of “excretion” and toxicities of the side-products. The redox potentials of different drugs, their dynamic concentrations in the liver cells and their logP/logD facets, their known affinities/mechanism based inhibitions of select enzymes if any, the extent of glycogen/fat deposits within the liver, etc. should be considered pivotal in a case-wise modeling of drug interactions involving CYPs. It is crucial to remember that not mere genetics govern metabolic predispositions in the liver, it is the reaction microenvironment that plays a greater role in CYPs' reactivity. More reliable liver models would be needed for phase I drug metabolism research in the times to come. In that regard, it is very important to profile the small molecules in liver microsomes/cells and gauge an impact of their activities on CYP mediated metabolism. No molecule sits as a spectator when a CYP goes to work. Quite simply, the CYP + CPR combo is too hot for that to happen! In an earlier communication, we had shown how the dynamics of the metabolism of a particular substrate (diclofenac) changes upon the incorporation of a “so-called inhibitor” (dihalophenolics) in two setups- baculosomes and microsomes (Parashar et al., 2014a). The pertinent data is presented in Supplementary Information, Table S2. It could be seen clearly therein that while benzbromarone and benziodarone inhibited both CYP2E1 and CYP2C9 in baculosome setups (containing only the respective isozymes as the functional agent), the more “realistic” microsome setups were activated (for diclofenac metabolism) upon the incorporation of at least some concentration range of these “arones.” Several small molecules activated the CYP2C9-diclofenac reactions at lower concentrations, when the same molecules served as inhibitors at equimolar concentrations. Quite evidently, the currently available theoretical understanding cannot be applied to generate inhibition constants obtained from *in vitro* studies (either pure reconstituted or baculosome systems) and such constants' relevance cannot be extrapolated to the complex functional roles in liver microsomes.

We support the statements above with a simple experiment. CYP2E1 and CYP1A2 can de-ethylate 7EFC (left panel of Figure S14, Supplementary Information), though the efficiency varies at different concentrations of the enzymes. When cholesterol was added to CYP1A2-7EFC, the IC_50_ values were ~1.03 μM and ~170 nM, respectively, at 2.5 μM and 5 μM EFC (right panel of Figure S14, Supplementary Information). The assay with 5 μM 7EFC (a concentration much higher than the supposed K_M_ of <0.1 μM; Code et al., 1997) afforded a K_i_ <3.5 nM (as calculated from the IC_50_ value, using Cheng-Prusoff equation; Yung-Chi and Prusoff, 1973) for cholesterol with CYP1A2. How could this be, when cholesterol shows (*in silico*) binding with an estimated K_d_ of ~7 μ M, a value which is several orders lower than the (*in silico*) binding affinity of 7EFC for the very same enzyme (CYP1A2)? This is counter-intuitive with respect to the prevailing ideas of binding-based inhibitions and is quite similar to inhibition of CYP2E1 by an agent like 4-methylpyrazole (Parashar and Manoj, submitted). Table S3 (Supplementary Information) shows that a heme-centered binding (docking) does not explain the observations. We have demonstrated that diffusible radical mediated inhibitions could afford such effects in P450/peroxidase systems (Parashar et al., 2014a,b). CYP2E1 is a heme-thiolate enzyme that does not have a readily accessible channel to the heme distal center (Porubsky et al., 2008), when the static structure was analyzed with PyMol. Recently, we had postulated and demonstrated that catalysis in these systems is mediated via diffusible species (Venkatachalam et al., 2016) and (Parashar and Manoj, submitted), Along the same lines, the crystal structure of CYP1A2 (Sansen et al., 2007) also does not show a readily accessible channel to the heme-center. So, it is only forthright to deduce that the mechanism of this enzyme also follows the same route as CYP2E1.

Further, it is noted that the much celebrated CYP inhibitor, aminobenotriazole (ABT) does not arrest enzyme activity by heme binding but causes a sort of “mechanism-based” inactivation (via the reactive benzyne formation; de Montellano et al., 1984). That is- ABT is an efficient substrate (nucleophile) whose oxidation leads to a reactive product that could diffuse and oxidize the heme- edges. It is somewhat similar to how a terminal olefin “mechanistically inhibits” CPO at high enzyme- additive concentrations (Debrunner et al., 1996). We had demonstrated that this inactivation is not seen when the enzyme is taken at nanomolar concentrations (Manoj and Hager, 2008). CYP2C9 is very poorly susceptible to ABT (Emoto et al., 2003) and this may be because its heme edges are not available for the reactive species. This inference is supported by CYP2C9's crystal structure (Williams et al., 2003). Therefore, much care should be exercised before attempting to device molecules for binding at the heme-center (aimed to inhibit catalysis at low concentrations of the enzyme).

Most substrates metabolized by a given CYP fail to induce the particular CYP. This seems quite rational, given their evolutionary mandate. Microsomal CYPs and the associated cellular machinery have no evolutionary “clue” about what substrate they would come across and would have to catalyze. So, nature produced a bevy of CYPs with diverse surface topographies and channel orientations. A substrate could either bind on the surface or interact through direct reduction. A diffusible radical stabilized within the heme pocket would emerge out and it could chance to meet the substrate molecule localized in or around the channel. The *murburn* hypothesis also explains why antibodies to a particular CYP can knock out the activity of that particular CYP in liver microsomal preparations. The *murburn* hypothesis explains both well-coupled and uncoupled P450 systems (*in vitro* or *in situ*). The proteins and substrates need to equilibrate before the reaction commences (and the dynamics of ROS varies into the reaction owing to the multiple equilibriums involved) and as a result, we could have a product forming at a higher rate after some time into an incubation too. (If some worker observes this, it should not be considered an artifact!) Hydrophobic proteins need several minutes of pre-incubation for effective catalysis. This means that the mobility and equilibration of these proteins is a very slow process, with respect to the overall kinetics.

## Conclusions

It is now opportune for us to move out of the “esthetic concern” that reactions involving reactive diffusible species (whether generated by CPR, indirectly produced in the milieu or formed by one-electron redox catalysis of the substrate) are chaotic. These are non-specific only when such reactive species are at much higher concentrations. For the critical reader, Table S4 of Supplementary Information presents an itemized comparison of the erstwhile hypothesis with the *murburn* concept. It can be seen that *murburn* concept is fully accommodative of all aspects known of CYP systems till date, whereas the erstwhile hypothesis fails to explain a bevy of observations and considerations. The *murburn* concept also explains the unique CPR's promiscuous ability to serve as electron donor to diverse CYPs and the versatility of CYPs (diverse xenobiotic substrates need not have high affinities for CYPs' heme-pockets for them to be reacted upon!). This manuscript ratifies several predictions/projections (Venkatachalam et al., 2016) of *murburn* hypothesis and lays foundations to some novel “cellular biochemistry” concepts:

- Aside their roles as molecular messengers and as disruptive agents in immunological responses against pathogens, diffusible reactive oxygen species (DROS, exemplified by superoxide radical) have been deemed as (patho)physiological manifestations of oxidative stress. The current work establishes that small amounts of diffusible radicals are generated *in situ* as an obligatory part of routine redox metabolism in liver microsomes. The findings confirm that reactive diffusible radicals like superoxide are involved as a catalytic requirement for routine housekeeping metabolism within cells. Such processes could even have been one of the major reasons for the success of the diradical molecular oxygen's evolution as a key protagonist in life processes, *per se*. Contrary to the prevailing thought paradigms, two sequential one-electron transfers may be quite 'normal' in cells. Electron transfers (for sustaining life and generating or stripping reducing electron-equivalents) in cells evolved obligatorily requiring the lipid fabric/platform to slow down the radical process. Further, proteins like Cyt. *b*_5_ are now understood to serve as steady-state “electronic buffers” in the phospholipid system. The fact that diffusible radical species (DROS, metabolites) are obligatorily involved in routine metabolism could explain both normal and idiosyncratic hepatotoxicity of drugs.
- Hitherto, enzymes are known to achieve rate enhancement (catalysis) via the formation of a “transition state” that entails a direct bond formation between the enzyme and reactant(s) OR between the reactants themselves. Cytochrome P450s catalyze xenobiotics' metabolism without invoking the erstwhile “transition state” concept (“lock and key”/“induced fit” theories). Herein, the reaction locus may be delocalized (uncertain) but is confined to the enzyme because it stabilizes the otherwise short-lived radicals.
- When redox equivalents are utilized for substrate hydroxylation in a well-coupled system, the oxygen-superoxide equilibrium gets shifted to the right, leading to the depletion of NADPH. In an uncoupled system, a similar tug is exerted by the formation of water from DROS. Therefore, the operating logic is a constitutive redox pull, same as that established in the peroxidase milieu (Manoj et al., 2016b).

We envisage that the findings and ideas revealed herein stand to usher in a new research paradigm in cellular redox biochemistry. The outcome of this work necessitates that new theoretical/quantitative approaches should be employed to address CYP reactions and also seeks that textbooks should be rewritten to abrogate the redundant ideas in the field.

## Author contributions

KMM planned the works, carried out experiments, analyzed results, proposed explanations and wrote the paper. SG, AV, and AP carried out experiments and reported data. AV and AP assisted in proofing the manuscript.

## Funding

The work was powered by Satyamjayatu: The Science and Ethics Foundation.

### Conflict of interest statement

The authors declare that the research was conducted in the absence of any commercial or financial relationships that could be construed as a potential conflict of interest.

## Abbreviations

4′OH or 4′OH Diclof: 4′hydroxydiclofenac
7EFC: 7-ethoxytrifluoromethylcoumarin
7HFC: 7- hydroxytrifluoromethylcoumarin
ABT: aminobenzotriazole
CPO: Chloroperoxidase
CPR: Cytochrome P450 Reductase
CYP: Cytochrome P450
Cyt. *b*_5_: Cytochrome *b*_5_
Cyt. *c*: Cytochrome *c*
Czx: Chlorzoxazone
Diclof: Diclofenac
DLPC: Dilauroyl Phosphatidyl Choline
(D)ROS: (Diffusible) Radicals and Reactive Oxygen Species
HRP: Horseradish peroxidase
NAD[P_H: Nicotinamide Adenine Dinucleotide (Phosphate) reduced]
Pdr: Putidaredoxin Reductase
pNP: p- Nitrophenol
rxn.: Reaction
SOD: Superoxide Dismutase
Vit C: Vitamin C
Vit E: Vitamin E
Warf: Warfarin.
