# Supplementary material for: Functioning of Microsomal Cytochrome P450s: Murburn Concept Explains the Metabolism of Xenobiotics in Hepatocytes
Source: Front Pharmacol. 2016 Jun 23;7:161. doi: 10.3389/fphar.2016.00161 (PMC4918403; doi:10.3389/fphar.2016.00161)
Supplement: Supplementary file 1 [file DataSheet1.pdf]

Supplementary Material

Functioning of microsomal cytochrome P450s:

Murburn concept explains the metabolism of xenobiotics in hepatocytes

Kelath Murali Manoj[1*], Abhinav Parashar[2], Sudeep K. Gade [2] & Avanthika Venkatachalam[3]

*** Correspondence: Kelath Murali Manoj (**Corresponding Author) satyamjayatu@yahoo.com

1.1 Supplementary Figures

Figure S1: a. Effect of substrate on peroxide formation in controlled setups containing CPR: Initial reaction conditions were: [NADH] = 180 µM, CPR 20 µl from 15-20 µM stock, total reaction volume 750 µl, 100 mM potassium phosphate buffer (pH 7.4), [substrate] = 100 µM and [lipid (DLPC)] = 50 µg/ml. **b: Effect of substrate on peroxide formation in controlled setups containing ferric citrate:** Reaction components at the commencement of reaction: [NADPH] = 150 µM, total reaction volume 300 µl, 100 mM potassium phosphate buffer(pH 7.4), [substrate] = 100 µM, [ferric citrate] = 10 nM.

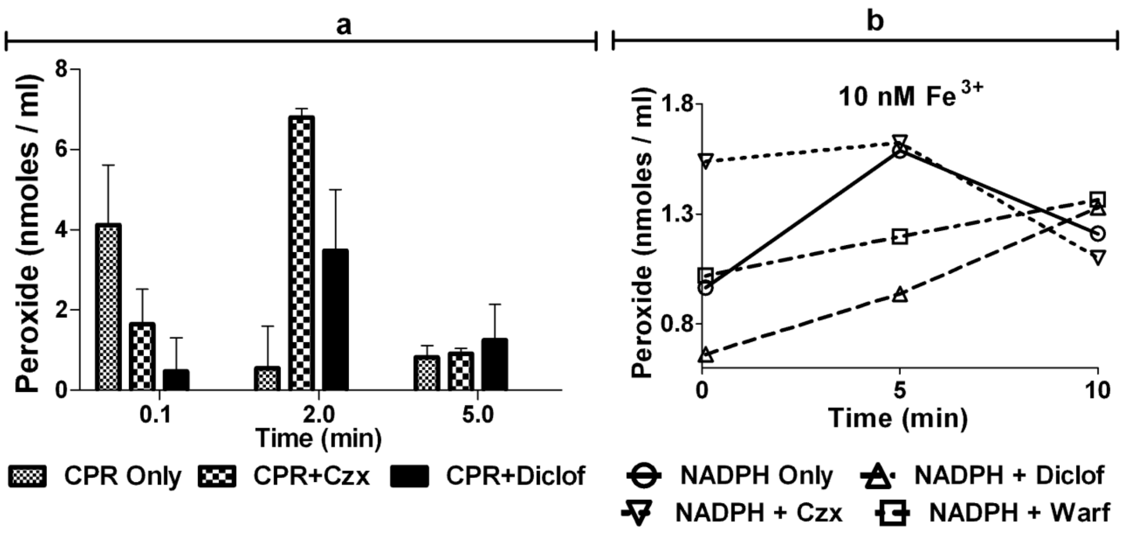

Figure S2: Peroxide profiles for reaction of select substrates with superoxide: Initial reaction mixture contained- substrates (Warf or Diclof) at 100 µM, [Superoxide] ~30 µM, 100 mM potassium phosphate buffer (pH 7.4), total reaction volume was 300 µl, temperature ~26 °C.

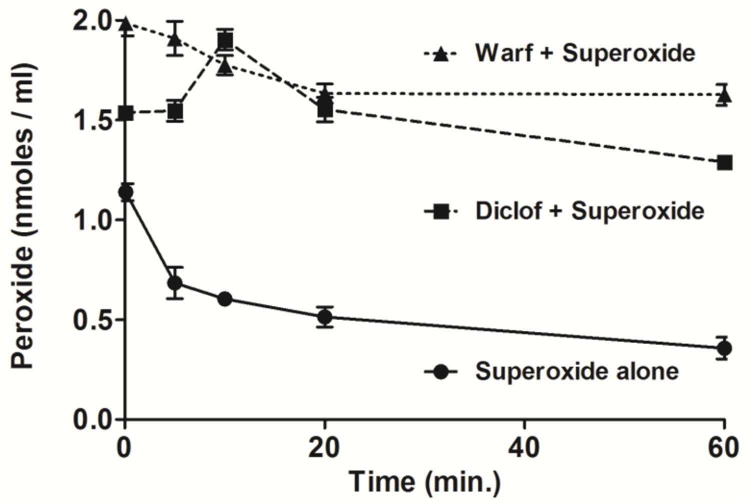

Figure S3: Demonstration of multiple roles of reaction components (in modulation of peroxide levels): Reactions lacking substrate- Initial conditions $[H_2O_2]$ = 100 µM, [NADPH] ~ 200 µM, [2C9] ~ 40 nM; [CPR] ~150 nM added after 15 min incubation, pH 7.4, 100 mM KPi, 37 ºC.

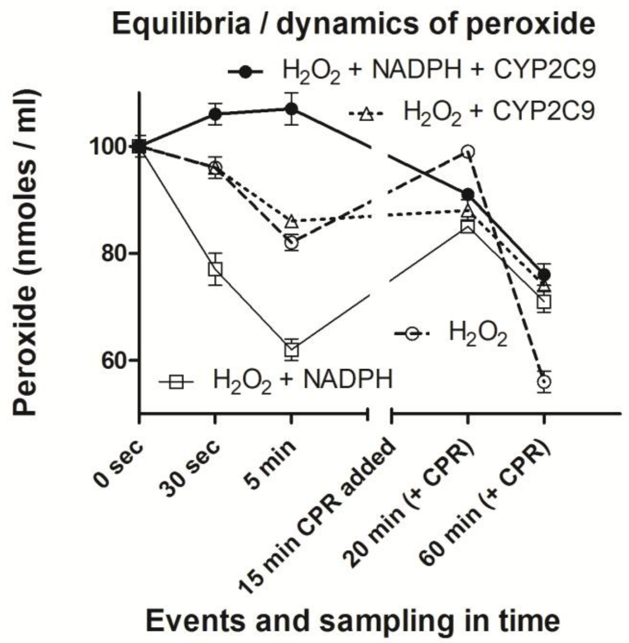

Figure S4: CPR's ability to deplete peroxide is affected by the inclusion of soluble ROS scavengers. The reaction mixture contained- 100 mM pH 7.4 potassium phosphate buffer, [H_2O_2] = 100 µM, [DBDA] & [tBC] = 10 mM, [CPR] = 250 nM, total reaction volume was 300 µl, ~ 26 °C. (DBDA, 4,5-dihydroxy-1,3-benzene-disulfonic acid; tBC, *tert*-butyl catechol)

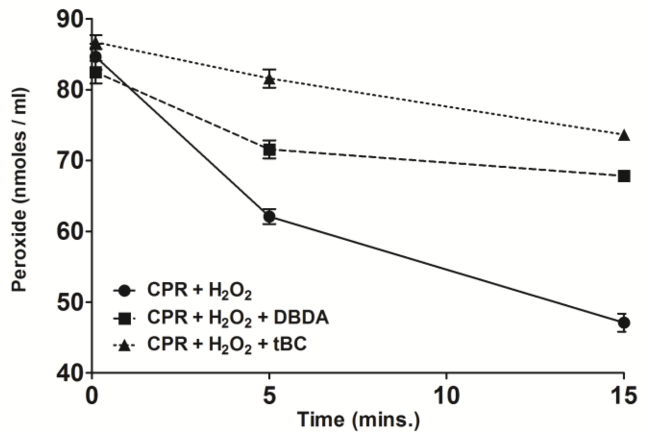

Figure S5: **Effect of redox-active additives:** Initial conditions [2C9] = 10 nM, [CPR] = 50 nM, [Diclof] = 200 µM, [redox additives] = 1 mM, [NADPH]=~250 µM.

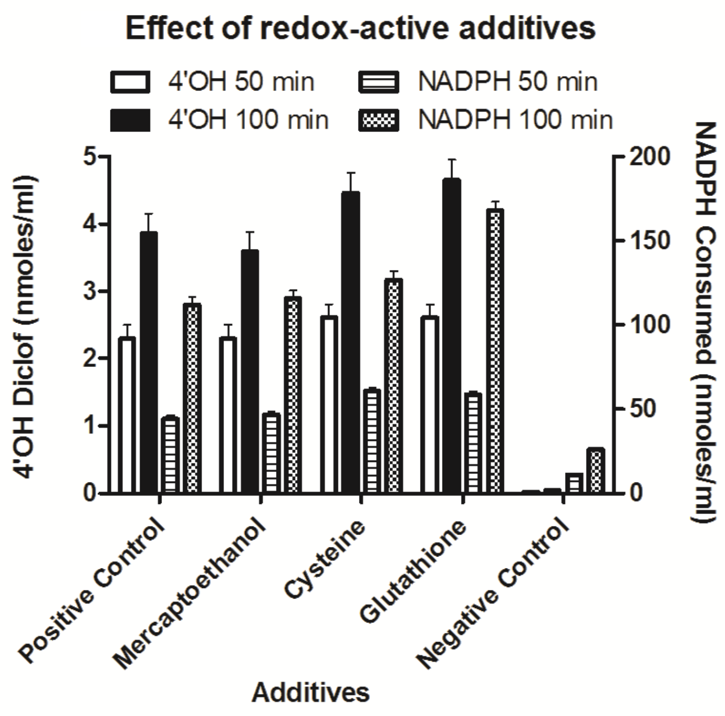

Figure S6: Redox active vitamins C & E inhibit cytochrome P450 reactions, showing temporal variations in the level of inhibition. The reaction was performed in 100 mM pH 7.4 potassium phosphate buffer, [Diclof] = 100 µM, [NADPH] = 200 µM, [Microsomes] = 0.5 µM, [CYP2C9] = 10 nM, Redox molecules were employed at 1 mM, total reaction volume was 300 µl, ~ 26 °C. [In a recent work (Parashar et al. 2014) we had reported the product formation by the incorporation of the very same ROS scavengers at 10 minutes. The same data is re-plotted herein and presented for a comparison with the 4-OH diclofenac profile obtained at 15 minutes. This is essential for understanding the dynamics of ROS within the reaction system. [We caution that the effects shown herein should not be dismissed with the statements that the system is uncoupled or the system is not in equilibrium. The fact that Figures S5, Figures 6 & 7 also demonstrated such effects is evidence that the data are not artifacts.] (LAAP, L-ascorbic acid palmitate)

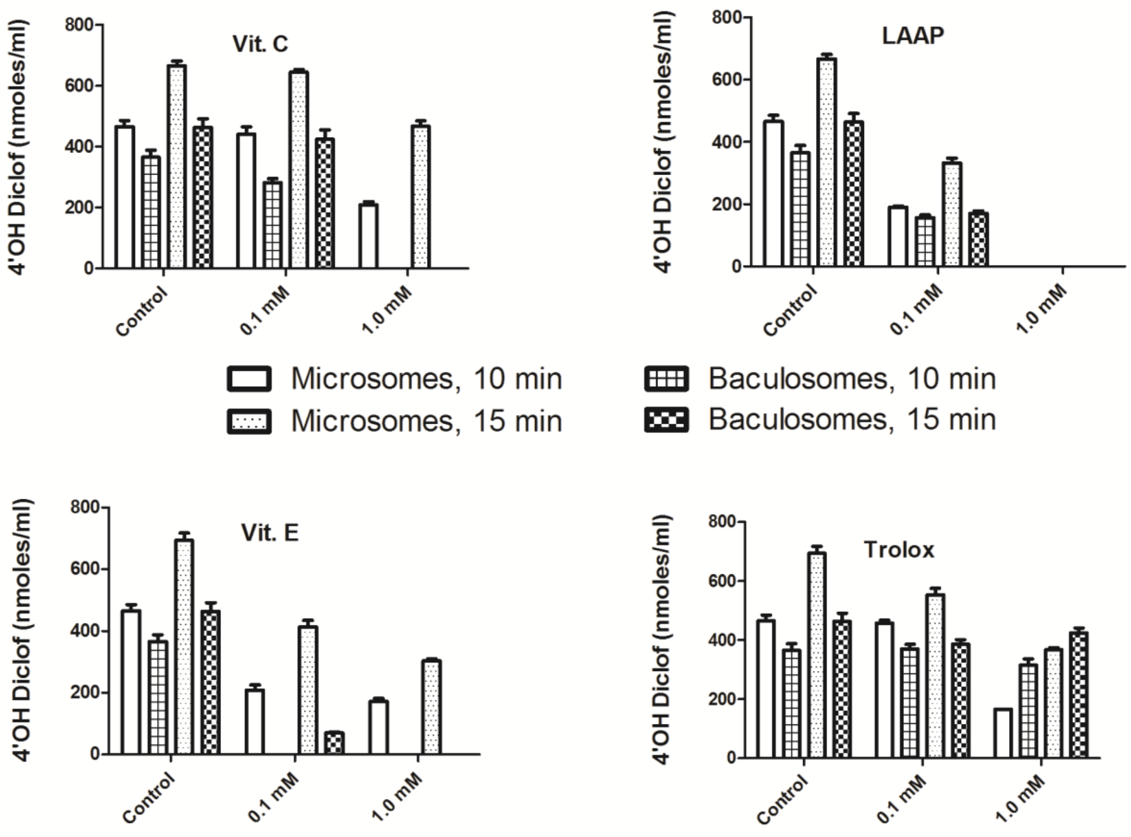

Figure S7: Standard HPLC chromatogram of diclofenac and its hydroxylation products is shown. Inset shows the product profiles of reaction incorporating DROS utilizing proteins. The chromatogram of reaction mixture with HRP did not give any measurable peaks within 6 to 6.5 minutes of elution time. The peaks eluting after 4'hydroxydiclofenac in the test reaction may also be the benzoquinone imine intermediate, not just another regiospecifically hydroxylated diclofenac.

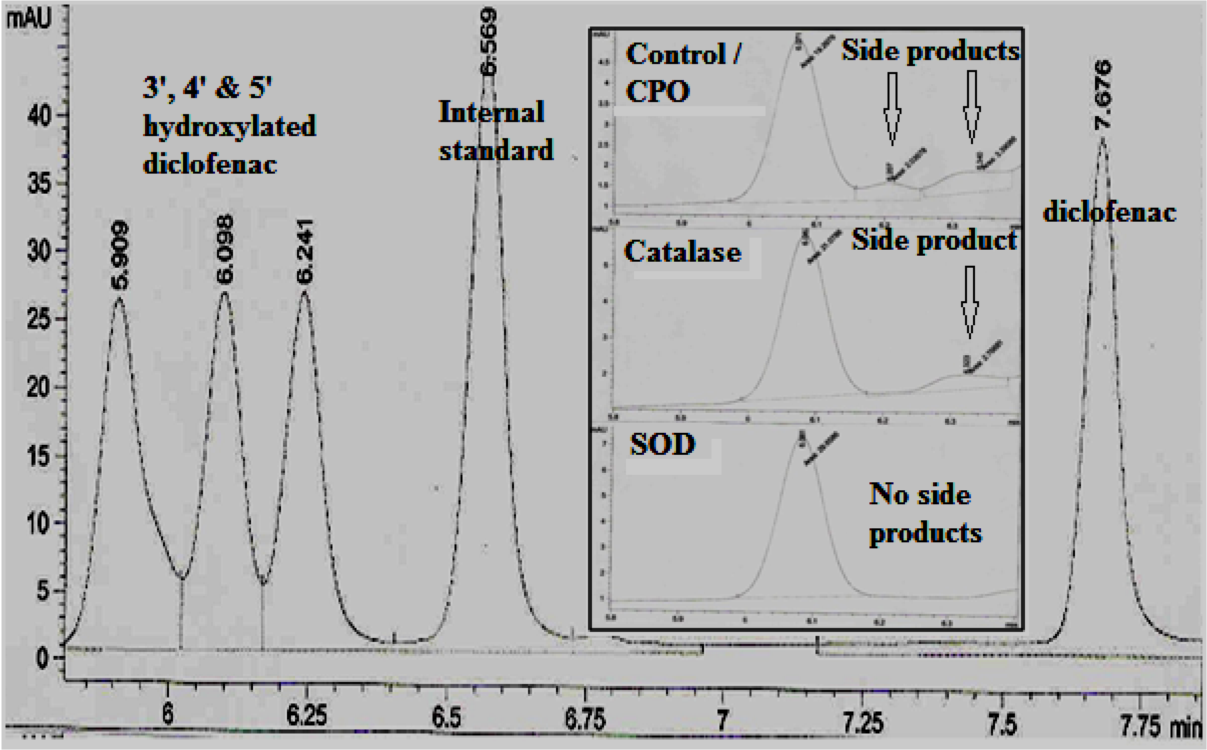

Figure S8: Effect of pH and CPO on CYP2C9 mediated hydroxylations: Initial conditions were- [NADPH] = 125 µM, [Diclof] = 100 µM, Invit.2C9 ~ 5 nM & CPO ~ 30 nM.

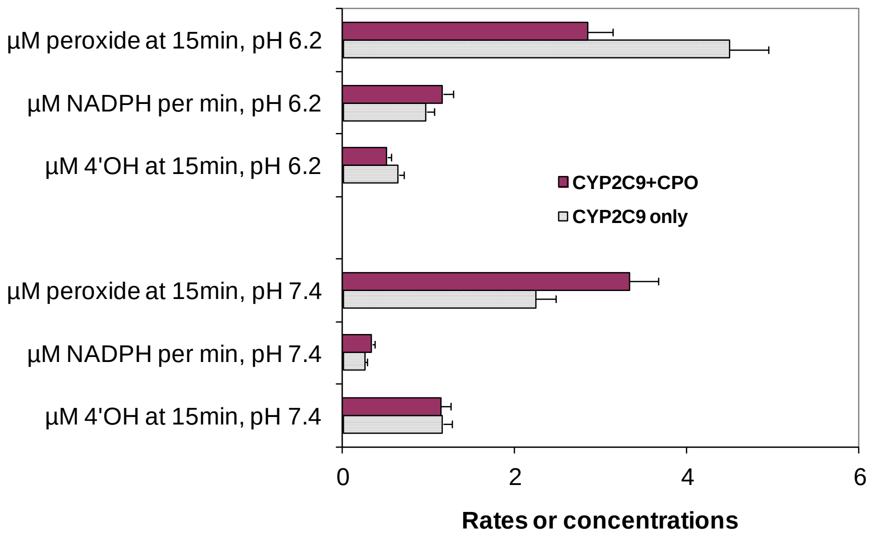

Figure S9: Monitoring NADPH and peroxide in controls for understanding the thermodynamic pull and multiple redox equilibria *in milieu*. The initial concentrations were [CPR] = 180 nM, [HRP] = 157 nM, [Diclof] = 200 µM & [NADPH] = 215 µM. The data points correspond to µM NADPH substrate consumed or µM peroxide *in milieu*.

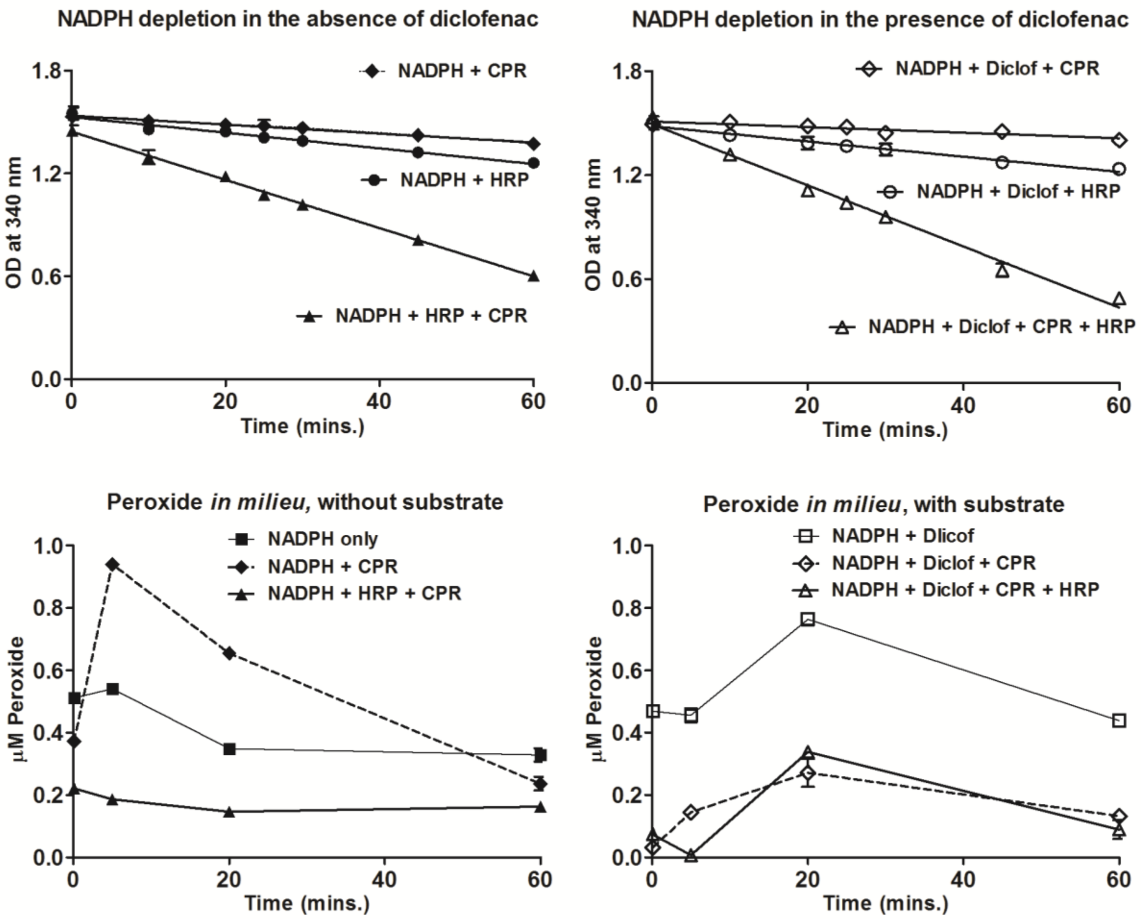

Figure S10: Spectra of HRP and probing its binding (experimental and *in silico*) with diclofenac. In the top panel, the slight lowering of the Soret OD (from trace 1 to 2) is fully accounted by the dilution. In the bottom panel, it can be seen that diclofenac binds far away from the heme and that the narrow channel of HRP is occluded for diclofenac's entry into the heme distal pocket.

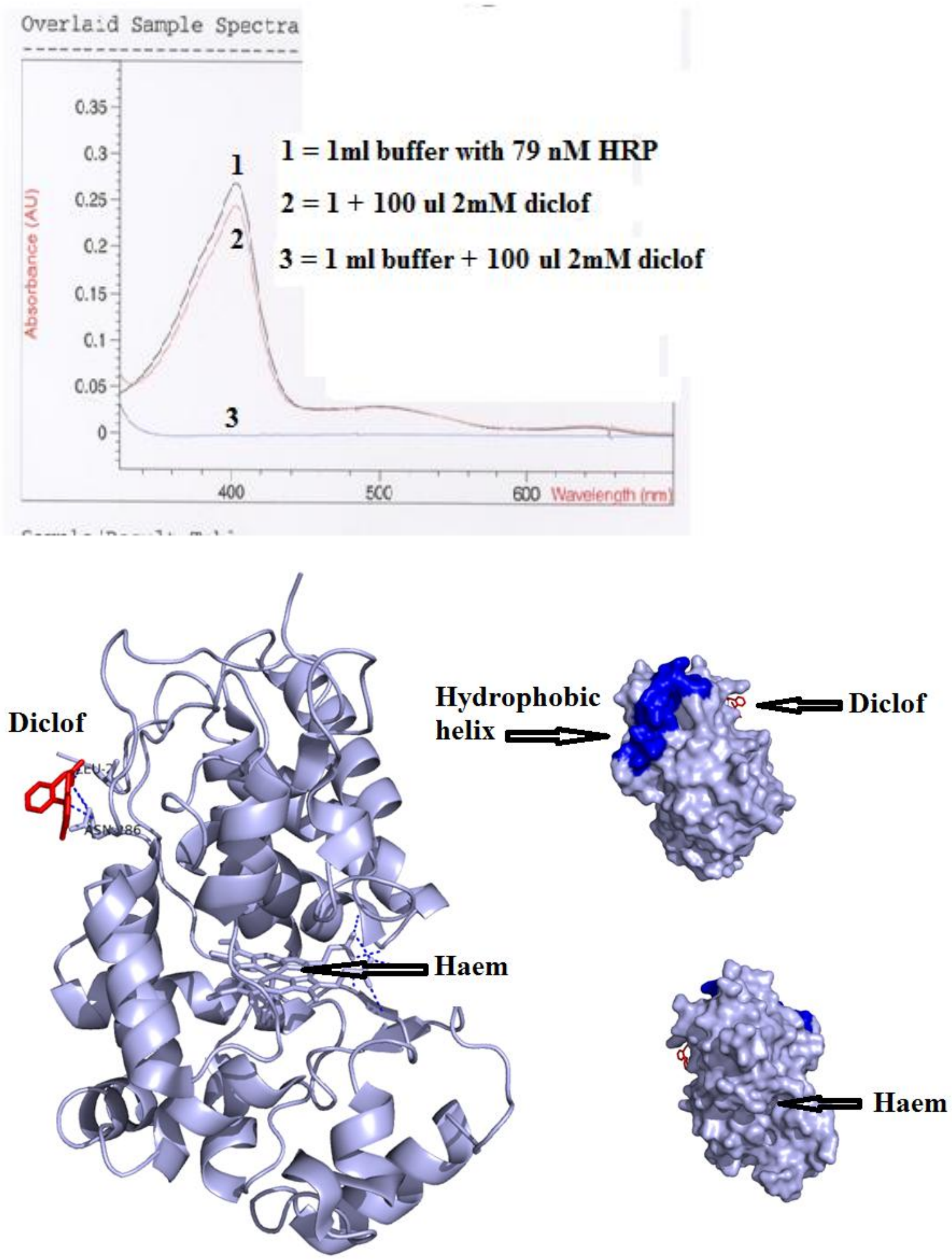

Figure S11: ***HPLC chromatograms of some CYP2C9 reactions.*** **(**The 4'hydroxylated diclofenac elutes at 6.36 minutes in these set of chromatograms, owing to a minor lowering of the organic phase component of the elution solvent mixture.**)** **Left panel:** Physical separation of CYP2C9 and CPR gave specific hydroxylation of diclofenac. *Profiles A & B* show the chromatograms of sample drawn at 45 minutes of reaction time from within the dialysis tubing and from the free solution in the test reaction respectively. In the test reaction, CYP and CPR were separated. Clearly, A shows the specific product formation. *Profile C* is the sample drawn from the positive control reaction in which CYP and CPR were mixed. *(Total reaction = 6 ml, 2 ml within tubing, [2C9] = 200 nM, [CPR] = 550 nM, [NADPH] = ~1.25 mM, [Diclof] = 200 µM)* **Center panel:** CYP2C9 catalyzed oxidation of hydroxydiclofenac. *Profiles M & N* **:** M is the sample drawn at 45 minutes of incubation and N is a sample drawn after 4 hours of reaction. The lowering of hydroxylated diclofenac peak and the appearance of side products (as a result of further oxidation of diclofenac) is seen at later time frame. Apparently, the 4'hydroxylated product was converted to more polar products as a result of which they eluted earlier (5.2 and 5.8 minutes). (*150 nM CYP2C9, 300 nM CPR, 2 mM NADPH & 200 µM Diclof*) **Right panel:** Verification of CYP2C9 catalyzed oxidation of hydroxydiclofenac. *Profiles X, Y & Z :* X is the control reaction which had 80 µM diclofenac only and it does not show any significant formation of the polar product. Y is the test reaction with only 40 µM of 4'hydroxydiclofenac and it shows the increased production of the more polar product. Z had both 40 µM 4'hydroxydiclofenac and 80 µM diclofenac added initially in the reaction mixture. As seen, the hydroxydiclofenac is formed to the same extent, irrespective of the presence of excess diclofenac. It shows that the latter is not an effective inhibitor in CYP2C9's oxidation of 4'hydroxydiclofenac. (*Incubations for 30 minutes had 6.8 nM Invitrogen CYP2C9 and 1.25 mM NADPH.*)

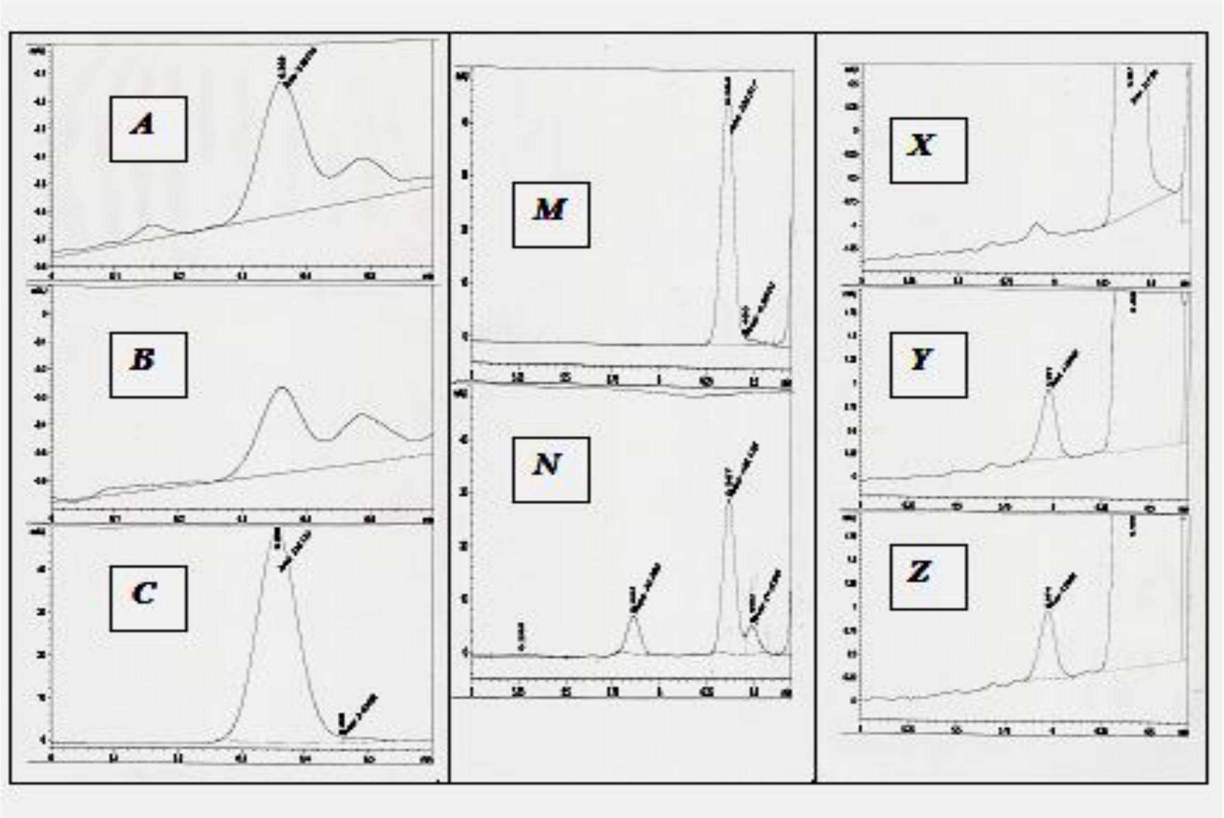

Figure S12: Specific hydroxylated product formation in different reaction conditions: Top panels (Expt. 1). Initial state: CYP2C9 = CPR ~ 50 nM, Cyt. b_5 if present, at 500 nM. **Middle panels (Expt. 2).** Initial state: CYP2C9 = 35 nM, CPR = 15 nM, Cyt. b_5 if present, at 20 or 200 nM. **Bottom panels (Expt. 3A)**. Initial state: CYP2C9 = 100 nM, 1mM NADPH, 300/1500 nM Cyt. b_5, no lipids; (**Expt. 3B).** Initial state: CYP2C9 = 100 nM, 1 mM NADPH.

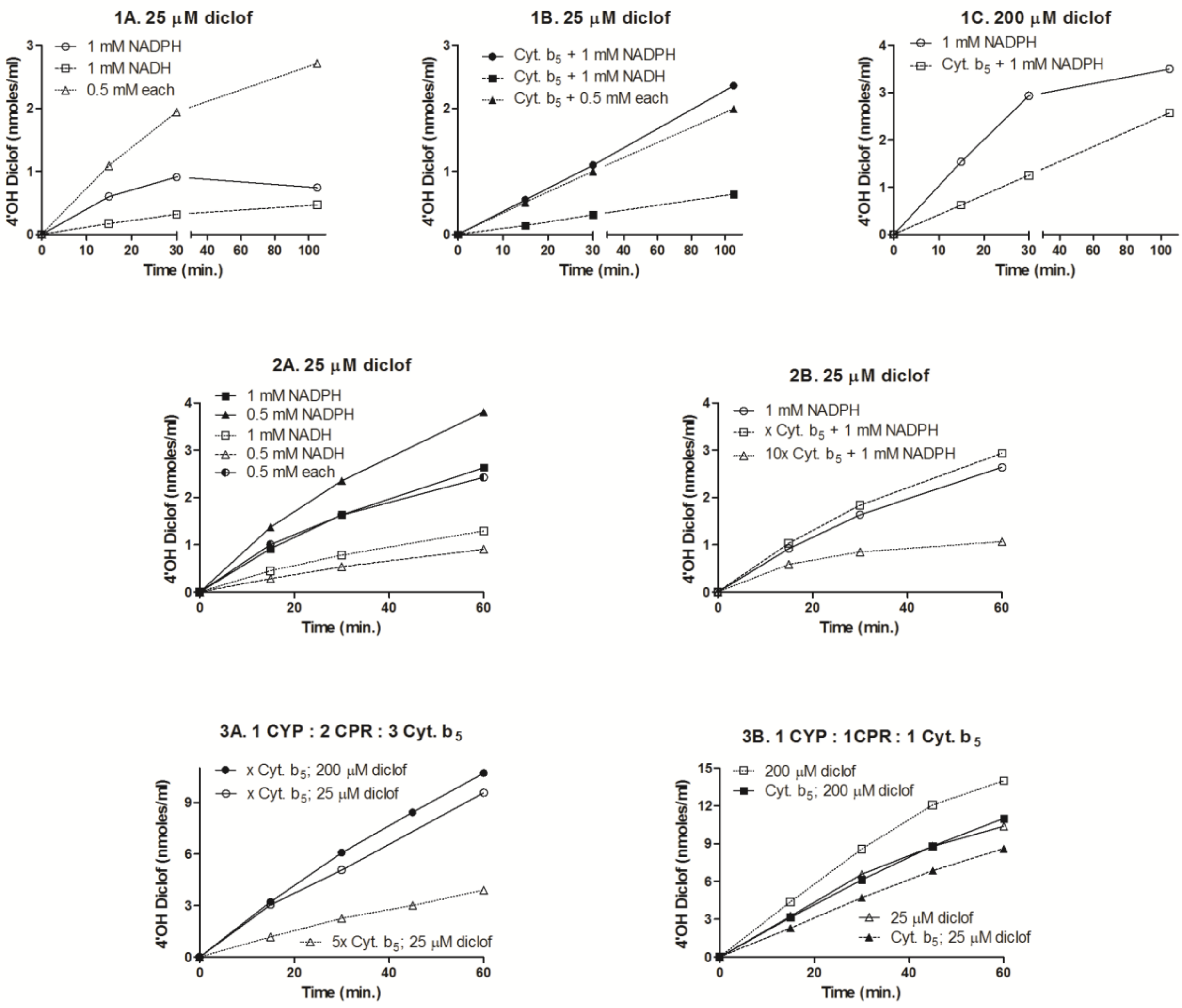

Figure S13: In reconstituted reactions, [CYP2C9] =190 nM, 660 nM crude CPR. In baculosomes, CYP2C9 concentration of 10 nM; [NAD(P)H] ~ 180 µM and [Diclof] = 200 µM. [Cyt. b_5]= 40 nM. In both these setups, initial rate of consumption of NADPH was lower for the reaction incorporating Cyt. b_5. But later on, the reactions with Cyt. b_5 show a higher rate of NADPH consumption. Generally, the reactions lacking Cyt. b_5 produced higher peroxide.

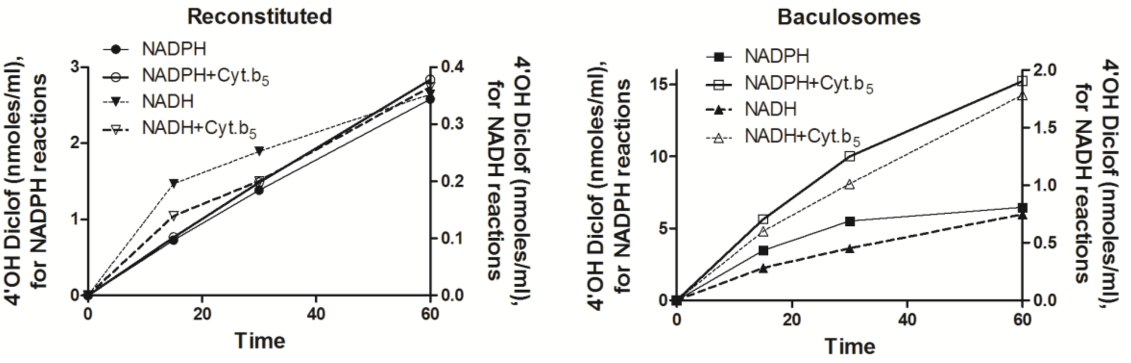

Figure S14: Comparison of CYP2E1 activity with CYP 1A2 and probing inhibition of CYP1A2 by cholesterol: Initial conditions were- Left panel: 310 µM NADPH, 200 µM CPR, 50 µM 7EFC, 20 µM DLPC. Right panel: [CPR] = 200 nM, [CYP1A2] = 100nM, [DLPC] = 20 µM, [NADPH] = 500 µM, [7EFC] = 2.5 & 5 µM, [Cholesterol] = 0, 0.1, 1 & 10 µM. The IC50 plot was fit with varying slope option.

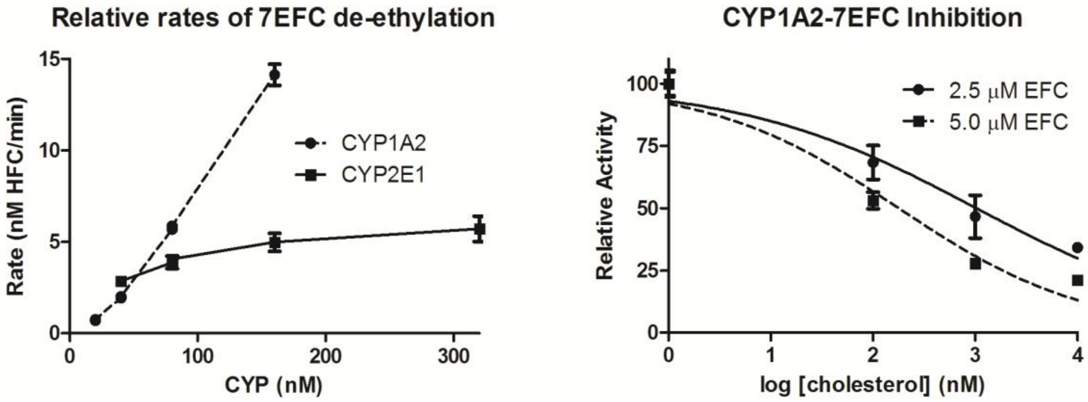

1.2 Supplementary Tables

Table S1: Prediction of trans-membrane hydrophobic helices in DROS modulating proteins: Several servers on the internet were used for trans-membrane segment prediction and the respective citations are given in the last column of the table. Reference codes of the proteins sequences used:- Human Cyt. b_5 - P00167, Rat Cyt b_5 - NP_071581, Rabbit Cyt. b_5 - NP_001164735.1, *A. rusticana* HRP - CCJ34838, human SOD - AAR21563, human Catalase - NP_001743, human Cyt. *c* - NP_061820.1, Rat Cyt. *c* - AAA21711.1, Horse Cyt. *c* - NP_001157486.

No.	**Algorithm**	**Cyt. b_5**	**HRP**	**SOD**	**Catalase**	**Cyt. *c***
1	waveTM	107 – 127	6 – 21	No trans-membrane segment predicted		
2	TMPred	109/10 – 126/7	3 – 21			
3	TopPred 1.10	107 – 127	3 – 23			
4	DAS	113 – 128	7 – 19			
5	TMHMM 2.0	109 – 131	5 – 27			
6	PRED-TMR	107 – 126	6 – 21			
7	SPLIT4	105 – 127/8	3 – 21			
8	TMMOD	109 – 129	3 – 23			
9	MPEx	109 – 131	7 – 19			
10	TOPCONS	109/10 – 129/30	5 – 27			
11	Predict-Protein	112/3 – 129/30	8 – 25			
12	PSI-PRED	110-131	4-23			
	**Consensus**	**109±4 to 129±2**	**5±2 to 24±3**			

Table S2. Comparison of dihalophenolics' inhibition parameters for CYP2C9-diclofenac in two systems: For each additive, the top row is the value for CYP2C9 baculosomes and the bottom row is the corresponding value for the mixed CYPs' microsomes. The value of K_M was taken to be 10 μM. All values are given in μM. (For details of additive structures/acronyms, please refer Parashar et al., 2014)

Additive	*IC_{50}*	*Std. dev.*	*R^2*	*K_i (non-linear)*	*Std. dev.*	*K_i (linear)*	*Std. dev.*	*R^2*
**Bzbr**	0.0052	0.0001	0.09	0.0005	0.00001	0.031	0.001	0.96
	-	-	-	-	-	-	-	-
**Bzr**	-	-	-	-	-	-	-	-
	-	-	-	-	-	-	-	-
**Bzir**	0.0305	0.002	0.97	0.0028	0.00016	1056	190	0.82
	-	-	-	-	-	-	-	-
**MeOBzbr**	-	-	-	-	-	-	-	-
	-	-	-	-	-	-	-	-
**MeOBzr**	-	-	-	-	-	-	-	-
	2.36	0.65	0.31	0.11	0.03	0.05	0.01	0.70
**MeOBzir**	-	-	-	-	-	-	-	-
	0.89	0.24	-1.38	0.04	0.01	98175	6872	0.93
**DIT**	468	163	-0.9	42.5	14.8	-	-	-
	240	100	-3.74	11.4	4.8	-	-	-
**DBP**	-	-	-	-	-	-	-	-
	-	-	-	-	-	-	-	-
**DBMP**	-	-	-	-	-	-	-	-
	-	-	-	-	-	-	-	-
**DBHBA**	1.0	0.4	-689	0.1	0.0	-	-	-
	0.67	0.32	-171	0.03	0.02	-	-	-

- *a non-entry means that at least one concentration activated the enzyme reaction in the given setup and therefore, it was not possible to calculate a global inhibition constant.*

Table S3: Docking of some ligands to select CYPs. 3E6I-pdb file was used for CYP2E1 & 2HI4- pdb file was used for CYP1A2.

**No.**	**Rigid CYP- Flexible ligand**	**Best binding parameters** ΔG (kcal/mol)	Distance (Å)	Amino acids	Orientation
**1**	CYP2E1-7EFC	-7.7	20.9	GLN 216, ASN 219	-
**2**	CYP1A2-7EFC	-10.3	6.0	PHE 226	+
**3**	CYP1A2-Cholesterol	-7.0	28.4	GLU 228, TYR 495	-

Table S4: An item-wise comparison of two explanations for CYP+CPR reaction system

Item	Issue	a. The erstwhile hypothesis	b. The 'murburn' concept	Which is discredited?
1	Presence of a CYP's substrate is not obligatory for the detection / observation of DROS in CYP+CPR reaction milieu. In the absence of substrate, NADPH is utilized.	Direct conflict! (Oxygen activation at heme distal pocket requires a Type I bound substrate.)	Agreeable.	a
2	In some CYP+CPR milieu, controls without substrates give higher DROS than the test reactions with substrate. Some substrates give higher peroxide in the CYP+CPR milieu than the control reactions lacking substrate.	Direct conflict with the first scenario! (Oxygen activation at heme distal pocket requires a Type I bound substrate.) Agreeable with second scenario.	Agreeable with both scenarios. (Controls show that superoxide's reaction with some substrates may give more peroxide.)	a
3	K_M values of some substrates for CYPs show high variability from lab to lab and across diverse reaction setups.	Direct conflict!	Agreeable.	a
4	Taking CPR separately in a dialysis membrane does not cease the electron transfers to proteins outside the membrane. When CYP is taken outside the membrane, the specific hydroxylation is noted in this scenario too.	Direct conflict!	Agreeable.	a
5	Peroxide profiles vary temporally in CYP+CPR reaction milieu. It may go up and then come down too (in a non-linear or even unidirectional way).	Direct conflict! (CYPs do not have significant peroxide utilization activity.)	Agreeable.	a

6.	Reaction stoichiometry varies temporally in CYP+CPR reaction milieu. Generally, the uncoupling leading to water formation increases in time. Significant loss of redox equivalents (non-accountable depletion of NADPH) is seen in several reactions.	Does not explain! (There is little reasoning to accept the formation of water at the heme-center by Compound I.)	Agreeable	a
7	Product formed could disappear in time and several secondary oxidations are observed.	Direct conflict! (CYPs are not supposed to have affinity for the hydroxylated substrate.)	Agreeable.	a
8	Several CYPs show lower product formation at higher concentrations of diverse substrates.	Poor explanation (that all these CYPs have multiple binding substrates for the diverse substrates) messes with Occam's razor!	Agreeable.	a
9	A unique CPR serves as electron donor to diverse CYPs of varying topologies.	Poor explanation (that all these CYPs have complementary electrostatic binding topology)!	Agreeable.	a
10	CPR is found at a distribution density of 1:10 to 1:100 wrt CYP.	Does not explain!	Explains.	a
11	Most CYPs and CPR work with or without their N-terms. Inclusion of CPR's broken N-term inhibits activity.	Not agreeable! (Advocates think that the N-terms are obligatory for protein-protein interactions.)	Agreeable.	a
12	CYPs have unusually high diversity in substrate utilization.	Explanation that all these are accommodated and	Agreeable.	a

		reacted at the unique heme distal pocket messes with Occam's razor!		
13	Inclusion of low amounts of SOD does not affect activity (but high amounts of SOD does inhibit).	Agreeable with the low amounts of SOD scenario only.	Agreeable. (If SOD is put in at higher amounts, it does inhibit the reaction!)	a
14	Inclusion of Vitamin E and Fatty acyl derivative of Vitamin C inhibits some CYP's activity whereas Trolox (the soluble derivative of Vitamin E) and Vitamin C do not.	Does not explain.	Explains.	a
15	Inclusion of catalytic amounts of HRP inhibits CYP activity. Inclusion of excess amounts of MetMb or MetHb does not significantly perturb activity.	Not agreeable!	Agreeable. (HRP has the ability to use both peroxide and superoxide and has a TMS region.)	a
16	CPR + NADPH gives DROS in reconstituted aerated samples.	Not agreeable!	Agreeable.	a
17	Intramolecular KIEs for aliphatic hydroxylation is large for several CYPs, implying that the molecule is free to rotate and unbound at the reaction center. In some cases, substrates of similar dimensions have smaller KIEs.	Direct conflict!	Agreeable.	a
18	Inclusion of cytochrome b_5 does not give a unidirectional rate enhancement for most CYP+CPR setups.	Does not agree!	Agreeable.	a

19	At a constant CYP concentration, increasing the CPR concentration (and measurement of product formation) gives a hyperbolic curve of relatively small amplitude at high CPR. This is when at the same constant CPR concentration; increasing the CYP concentration gives a sigmoid curve with much greater rate at high CYP.	Does not agree!	Agreeable.	a
20	Electron transfers and hydroxylations can be obtained with non-conventional redox partners.	Not agreeable!	Agreeable.	a
21	Low levels of redox-active additives (ions, small molecules, enzymes) can inhibit ET rates by CPR.	Direct conflict!	Agreeable.	a
22	A Type I binding spectra is not obtained with several substrates of diverse CYPs.	Direct conflict!	Agreeable.	a
23	Why is CYP3A4 so active? Why are the major CYPs more active than the rest? Why does CYP2E1 need Cyt. b_5?	Cannot explain.	Explains.	a
24	Fundamentals of molecular dynamics and diffusion kinetics	Direct conflict!	Agreeable.	a
25	Bizarre small values of constants (like K_M, IC_{50}, K_i)	Direct conflict!	Agreeable.	a
26	In the available crystal structures, most substrates bound with CYPs are positioned too far away from the heme center for a direct bond formation at the heme.	Direct conflict!	Agreeable.	a
27	Available crystal structures do not explain specificity (or high reaction rate) towards substrate and selectivity in terms of regio-	Does not agree!	Affords greater scope to explain.	a

	and stereo- aspects.			
28	Hydroxylations of too large a molecule (at seemingly inaccessible loci), something which cannot be accommodated even by large scale opening of F & G loops of CYPs	Does not explain!	Explains.	a
29	How do diverse amino acid residue mutations affect catalytic activity of CYPs, particularly when some of them are located at the surface of the CYP?	Does not explain!	Explains.	a
30	How can we explain mechanism based inactivation (by covalent modification of surface amino acid residues) by substrate molecules?	Does not explain!	Explains.	a
31	Why does a small topographical or moiety change in the substrate change reactivity of a substrate (within a class of molecules)?	Does not explain!	Explains.	a
32	Why do structurally similar (wrt to heme distal pocket) CYPs give different substrate preferences? (Else- Why do structurally different CYPs give similar substrate preferences?)	Does not explain!	Explains.	a
33	Atypical kinetics (experimental points that do not agree with a Michaelis-Menten paradigm)	Does not explain!	Explains.	a
34	Unpredictably mixed inhibition and activation profiles	Does not explain!	Explains.	a
35	Idiosyncratic and hormetic dose responses (activations or inhibitions)	Does not explain!	Explains.	a
36	Loss of NADPH redox equivalents, leading to the formation of water and	Does not explain!	Explains.	a

	peroxide, in the presence of substrate.			
37	Drug-drug interactions: A molecule can be an activator and an inhibitor of the very same CYP activity, with respect to a substrate (depending on concentration) or diverse number of substrates.	Does not explain!	Provides scope for explanation.	a
38	Multiple catalytic species (some of which are also capable of reduction), etc.	Does not explain!	Explains.	a
39	Zeroth order dependence (until low micromolar levels) on NADPH concentrations for CYP-CPR reactions and CPR mediated ET rates	Does not explain!	Explains.	a
40	High ionic strength impacts the rate of ET in CPR reactions	Does not explain!	Explains.	a
41	The extent of inhibition of ET in CPR reactions by hydrophilic or hydrophobic one-electron scavengers is dependent on the addition/presence of lipids.	Does not explain!	Explains.	a
42	Low yields (when compared to cytochrome c) of reduced cytochrome b_5 (which is hydrophobic and has a much favorable redox potential than P450s) in CPR mediated ETs	Does not agree!	Provides room for explanation.	a
43	CYPs can efficiently hydroxylate substrates with stabilized superoxide alone, without the need for CPR.	Does not agree!	Agrees.	a
44	When a CYP can kinetically differentiate between the R and S enantiomer of a substrate, why is the substrate not hydroxylated with high enantioselectivity?	Does not agree!	Explains.	a

45	Generally, CYP mediated hydroxylations are seen on the energetically favorable carbon atom and not the sterically unhindered carbon atom. Oxygen insertions on small molecules with activated carbon or heteroatoms are stereoselective but large molecules that lack such activated moities are seldom hydroxylated enantioselectively.	Does not explain!	Explains.	a
46	*In silico* explorations of substrate binding at the distal heme pocket gives substrate binding with very unfavorable orientations and distances.	Does not agree!	Agrees.	a
47	*In silico* binding of substrate and products with CYPs' distal heme pockets give comparable binding energies and orientations.	Does not agree!	Explains.	a
48	Substrate-bound and substrate-free crystal structures of CYPs do not show significant differences.	Does not explain the "induced fit" angle.	Agrees.	a
49	*In silico* probing shows that some large drug molecules bind more efficiently with surface crypts than within the heme distal pocket. In certain cases, substrates show a positive binding energy term at the heme distal pocket, implying that the binding is highly unfavorable.	Does not agree!	Agrees.	a
50	*In silico* probing of some small drug molecules show better binding at the distal heme pockets of CYPs but these molecules are not converted in actual reaction mixtures.	Does not agree!	Agrees.	a
